# The p38/HOG stress-activated protein kinase network couples growth to division in *Candida albicans*

**DOI:** 10.1371/journal.pgen.1008052

**Published:** 2019-03-28

**Authors:** Adnane Sellam, Julien Chaillot, Jaideep Mallick, Faiza Tebbji, Julien Richard Albert, Michael A. Cook, Mike Tyers

**Affiliations:** 1 Infectious Diseases Research Centre (CRI), CHU de Québec Research Center (CHUQ), Université Laval, Quebec City, QC, Canada; 2 Department of Microbiology, Infectious Disease and Immunology, Faculty of Medicine, Université Laval, Quebec City, QC, Canada; 3 Institute for Research in Immunology and Cancer (IRIC), Department of Medicine, Université de Montréal, Montréal, Québec, Canada; 4 Department of Medical Genetics, University of British Columbia, Vancouver, British Columbia, Canada; 5 Centre for Systems Biology, Lunenfeld-Tanenbaum Research Institute, Mount Sinai Hospital, Toronto, Canada; University College Dublin, IRELAND

## Abstract

Cell size is a complex trait that responds to developmental and environmental cues. Quantitative size analysis of mutant strain collections disrupted for protein kinases and transcriptional regulators in the pathogenic yeast *Candida albicans* uncovered 66 genes that altered cell size, few of which overlapped with known size genes in the budding yeast *Saccharomyces cerevisiae*. A potent size regulator specific to *C*. *albicans* was the conserved p38/HOG MAPK module that mediates the osmostress response. Basal HOG activity inhibited the SBF G1/S transcription factor complex in a stress-independent fashion to delay the G1/S transition. The HOG network also governed ribosome biogenesis through the master transcriptional regulator Sfp1. Hog1 bound to the promoters and cognate transcription factors for ribosome biogenesis regulons and interacted genetically with the SBF G1/S machinery, and thereby directly linked cell growth and division. These results illuminate the evolutionary plasticity of size control and identify the HOG module as a nexus of cell cycle and growth regulation.

## Author summary

The basis for commitment to cell division in late G1 phase, called Start in yeast and the Restriction Point in metazoans, is a critical but still poorly understood aspect of eukaryotic cell proliferation. All eukaryotic cells must grow to a critical cell size before commitment to division occurs. This size threshold couples cell growth to division and thereby establishes long-term size homeostasis. Here, to address the problem of cell size control across different species, we performed the first quantitative survey of the size phenome in the pathogenic yeast *Candida albicans* by focusing on mutants disrupted for protein kinases and transcriptional regulators. We investigate one of the divergent size regulatory pathways in *C*. *albicans*, the p38/HOG MAPK module, to uncover a novel stress-independent function for the HOG module in size control. We show that Hog1 inhibits G1/S transcription to delay the G1/S transition and that Hog1 also controls ribosome biogenesis gene expression. These results suggest that Hog1 represents a critical and previously unsuspected nexus between the growth and division machineries. Our study delineates the overall genetic basis for size control in *C*. *albicans* and identifies, to our knowledge, the first specific conduit that couples cell growth to cell division.

## Introduction

A central and longstanding problem in cell biology is how cells maintain a uniform cell size, whether in single-celled organisms or in the multitude of tissues of metazoans [1, 2]. In most eukaryotes, attainment of a critical cell size is necessary for commitment to cell division in late G1 phase, called Start in yeast and the Restriction Point in metazoans. This critical cell size threshold coordinates cell growth with cell division to establish a homeostatic cell size [1]. The dynamic control of cell size facilitates adaptation to changing environmental conditions in microorganisms and therefore is essential to maximize fitness [3, 4]. In the budding yeast *Saccharomyces cerevisiae*, the size threshold is dynamically modulated by nutrients. Pre-Start G1 phase cells grown in the optimal carbon source glucose pass Start at a smaller size if shifted to glycerol, whereas cells shifted from a poor to rich nutrient source pass Start at a larger size [1, 5]. Nutrient conditions similarly dictate cell size in the fission yeast *Schizosaccharomyces pombe*, although control is primarily exerted at the G2/M transition [5]. In metazoans, cell size control is important for tissue, organ and organism size [6], and is dynamically regulated through changes in growth rate and cell cycle length [7]. Cell size is often perturbed in human disease, for example in diabetes, tuberous sclerosis, mitochondrial disorders, aneuploid syndromes, cancer, and aging [1, 8]. Notably, a loss of cell size homeostasis, termed pleomorphism, correlates with poor cancer prognosis [9].

Cell size is fundamentally dictated by the balance between cell growth and division. The analysis of small-sized mutants in yeast led to key insights into the cell division machinery [10–14]. In all eukaryotes, cell division is controlled by the cyclin dependent kinases (CDKs), which serve to coordinate the replication and segregation of the genome [15]. In *S*. *cerevisiae*, the G1 cyclins Cln1, Cln2 and Cln3 trigger Start, whereas the B-type cyclins Clb1-Clb6 catalyze replication and mitosis, all via activation of the same Cdc28 kinase catalytic subunit. The expression of ~200 genes at the end of G1 phase, most vitally *CLN1*/2, is controlled by transcription factor complexes composed of Swi4 and Swi6 (SBF), and Mbp1 and Swi6 (MBF). Activation of SBF/MBF depends primarily on the Cln3-Cdc28 kinase, the key target of which is Whi5, an inhibitor of SBF/MBF-dependent transcription [16, 17]. Another transcriptional inhibitor called Nrm1 specifically inhibits MBF after Start but does not cause a marked size phenotype under conditions of nutrient sufficiency [17]. Size control in *S*. *pombe* is exerted through inhibition of G2/M phase CDK activity by the Wee1 kinase, which is encoded by the first size control gene discovered [10, 12]. Size is also partially regulated at Start in *S*. *pombe* through an SBF/MBF- like G1/S transcription factor complex and the Nrm1 inhibitor [18]. The CDK-dependent control of G1/S transcription in metazoans is analogously mediated by the cyclin D-Rb-E2F axis [16, 19, 20].

Cell growth depends on the coordinated synthesis of protein, RNA, DNA and other macromolecules [1, 21, 22]. The production of ribosomes consumes a large fraction of cellular resources and depends on an elaborate ribosome biogenesis machinery [1] that is controlled in part by the conserved TOR (Target Of Rapamycin) nutrient sensing network [6]. In budding yeast, the deletion of ribosome biogenesis (*Ribi*) factors causes a small cell size, and loss of two master regulators of *Ribi* gene expression, the transcription factor Sfp1 and the AGC kinase Sch9, causes cell to become extremely small [23]. These observations lead to the hypothesis that the rate of ribosome biogenesis is one metric that dictates cell size [24]. Sfp1 and Sch9 are critical effectors of the TOR pathway and form part of a dynamic, nutrient-responsive network that controls the expression of *Ribi* and ribosomal protein (*RP*) genes [24]. Sfp1 activity is controlled through its TOR-dependent nuclear localization [23–26] and is physically linked to the secretory system by its interaction with the Rab escort factor Mrs6 [25, 27]. Sch9 is phosphorylated and activated by TOR, and in turn inactivates a cohort of repressors of *RP* genes called Dot6, Tod6 and Stb3 [28]. The TOR network also controls size in *S*. *pombe* and metazoans [29].

Systematic genetic analyses in various species have uncovered hundreds of genes that directly or indirectly affect cell size. Direct size analysis of all strains in the *S*. *cerevisiae* gene deletion collection uncovered a number of potent size regulators, including Whi5, Sfp1 and Sch9 [23, 30, 31], and revealed inputs into size control from ribosome biogenesis, mitochondrial function and the secretory system [24–27]. Subsequent analyses of many of these size mutants at a single cell level have suggested that the critical cell size at Start may depend on growth rate in G1 phase and/or on cell size at birth [32, 33]. Visual screens of *S*. *pombe* haploid and heterozygous deletion collections for size phenotypes also revealed dozens of novel size regulators, many of which altered size in a genetically additive fashion [34, 35]. Many genes also influence size in metazoan species. A large-scale RNAi screen in *Drosophila melanogaster* tissue culture cells revealed hundreds of genes as candidate size regulators, including known cell cycle regulatory proteins [36]. Despite overall conservation of the central processes that control cell growth and division, functionally equivalent size regulators are often not conserved at the sequence level. For example, the G1/S transcriptional regulators SBF/MBF and Whi5 bear no similarly to the metazoan counterparts E2F and Rb, respectively [1]. A number of TOR effectors are also poorly conserved at the sequence level, including the ribosome biogenesis transcription factors Sfp1 in yeast and Myc in metazoans [1].

*Candida albicans* is a diploid ascomycete yeast that is a prevalent commensal and opportunistic pathogen in humans. *C*. *albicans* is a component of the normal human flora, colonizing primarily mucosal surfaces, gastrointestinal and genitourinary tracts, and skin [37]. Although most *C*. *albicans* infections entail non-life-threatening colonization of surface mucosal membranes, immunosuppressed patients can fall prey to serious infections, such as oropharyngeal candidiasis in HIV patients and newborns, and lethal systemic infections known as candidemia [38]. Interest in *C*. *albicans* is not limited to understanding its function as a disease-causing organism, as it has an ecological niche that is obviously distinct from the classic model ascomycete *S*. *cerevisiae*. In this regard, *C*. *albicans* has served as an important evolutionary milepost with which to assess conservation of biological mechanisms, and recent evidence suggests a surprising extent of rewiring of central signalling and transcriptional networks as compared to *S*. *cerevisiae* [39–43].

In this study, we performed a quantitative analysis of gene deletion mutants from different collections of protein kinases and transcriptional regulators in *C*. *albicans*. Our results revealed a noticeable degree of divergence between genes that affect size in *C*. *albicans* versus *S*. *cerevisiae* and uncovered previously undocumented regulatory circuits that govern critical cell size at Start in *C*. *albicans*. In particular, we delineate a novel stress-independent function of the p38/HOG MAPK network in coupling cell growth to cell division. Our genetic and biochemical analysis suggests that the HOG module directly interacts with central components of both the cell growth and cell division machineries in *C*. *albicans*.

## Results

### Analysis of the cell size phenome in *C*. *albicans*

**Fig 1:**
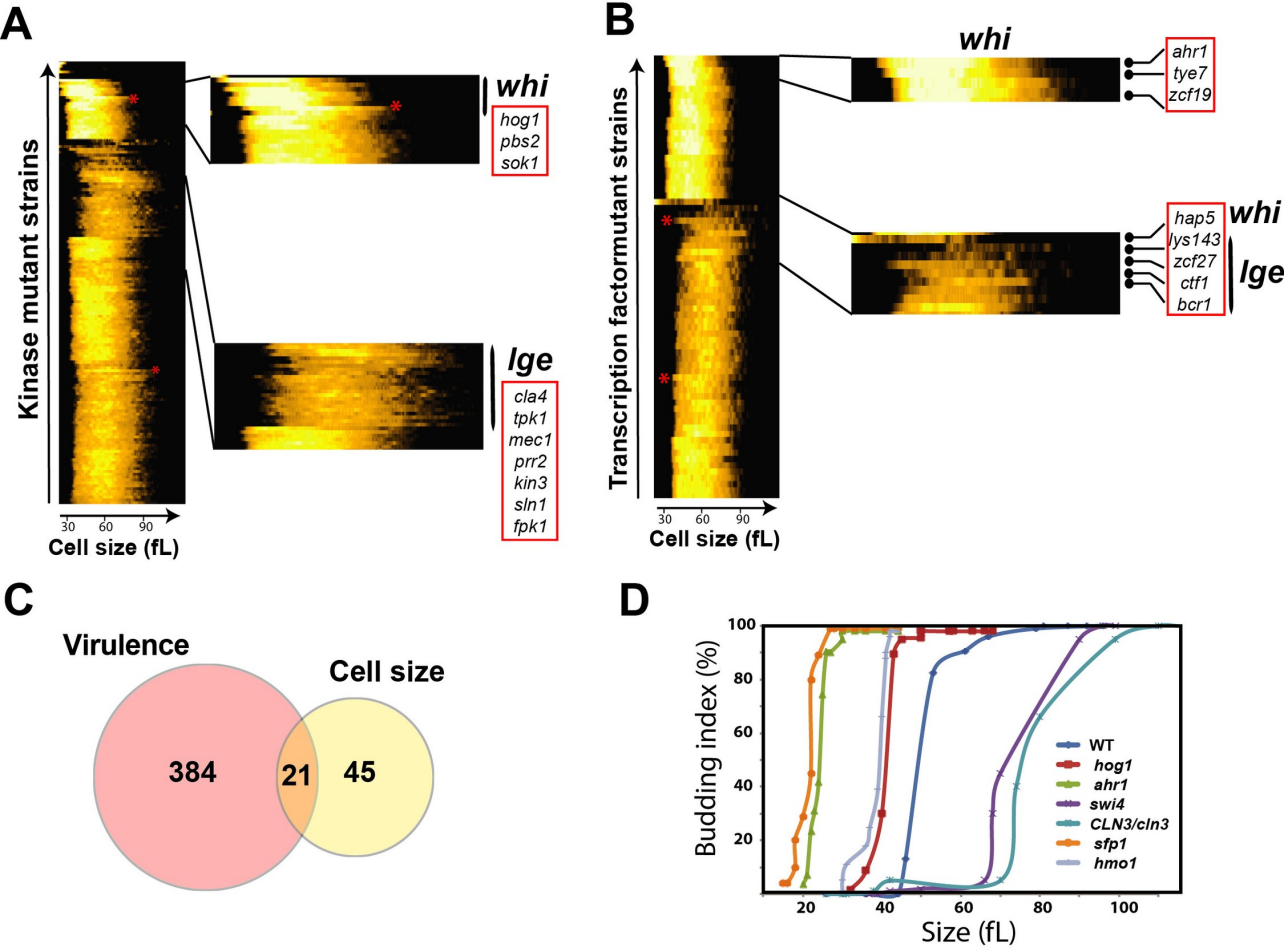
The cell size phenome of *C*. *albicans*. Clustergrams of size profiles of two different systematic mutant collections of *C*. *albicans*. (**A**) a set of 81 kinases [41] and (**B**) a set of 166 transcription factors [42]. For each strain, the cell volume distribution in femtoliters (fL) was measured over 256 size bins with a Beckman Coulter Z2 Channelizer. Hierarchical clustering was used to self-organize the datasets. Red asterisk in the clustergrams indicates size distribution of wt strains. Sections of the clusters corresponding to small and large size mutants are magnified. (**C**) Overlap between *C*. *albicans* size and virulence phenotypes. Avirulent mutant phenotypes were obtained from CGD based on decreased competitive fitness in mice and/or reduced invasion and damage to host cells. (**D**) Size regulators in *C*. *albicans* act at Start. Early G1-phase cells of different size mutant and wt (SN250 [47]) strains were isolated by centrifugal elutriation, released into fresh YPD medium and monitored for bud emergence and cell size at 10 min intervals until the entire population was composed of budded cells. Budding index was determined as the percentage of budded cells in each sample for at least 200 cells.

The diploid asexual lifestyle of *C*. *albicans* complicates loss-of-function screens because both alleles must be inactivated to reveal a phenotype unless gene function is haploinsufficient [44]. To identify genes required for cell size homeostasis in *C*. *albicans*, we directly screened three collections of homozygous diploid gene deletion strains that encompassed 202 transcriptional regulators [42, 45] and 77 protein kinases [41]. We expected transcription factors and kinases to be enriched in cell size regulatory genes based on previous studies in budding yeast and fission yeast. We additionally examined selected homozygous deletion strains of *C*. *albicans* orthologs of known size genes in *S*. *cerevisiae* (*sch9*, *pop2*, *ccr4* and *nrm1*; note that lower case gene names are used to indicate a homozygous mutant) that were not present in these deletion collections (**S1 Table**). In total, 363 viable mutant strains (279 unique mutants) were individually assessed for their size distribution under conditions of exponential growth in rich medium. Clustering of size distributions across the cumulative datasets revealed distinct subsets of both large and small mutants, relative to the majority of mutants that exhibited size distributions comparable to those of wild-type (wt) control strains (**Fig 1A and 1B**). Mean, median and mode cell size were estimated for each mutant strain, and mutants were classified as large or small on the basis of a stringent cut-off of a 20% increase or decrease in median size as compared to the parental strain background. This empirical cut-off value was determined based on a benchmark set of conserved small (*sch9*, *sfp1*) and large (*swi4*, *pop2*, *ccr4*) sized mutants for which median size was reduced or increased at least 20% as compared to parental strains. Based on this criterion, we identified 66 mutants that exhibited a size defect compared to their parental strain, comprised of 32 small-sized mutants (which we refer to as Whi phenotype, after the "Whiskey" designation used for the first known *S*. *cerevisiae* size mutants, [46]) and 34 large-sized mutants (referred to as a Lge phenotype [22]) (**S2 and S3 Tables**).

Deletion mutants of the HOG MAPK pathway (*hog1*, *pbs2*) and the morphogenesis checkpoint kinase (*swe1*) resulted in a small cell size phenotype. Conversely, mutants defective in functions related to the G1/S phase transition (*swi4*, *ace2*), filamentous growth and nitrogen utilization (*gat1*, *gzf3*, *dal81*, *rob1*) caused a large cell size phenotype (**S1B and S1C Fig**). As in *S*. *cerevisiae*, disruption of the central SBF (Swi4-Swi6) G1/S transcription factor complex increased cell size, whereas mutation of the ribosome biogenesis regulators Sch9 and Sfp1 reduced cell size, as did inactivation of Cbf1, the major transcriptional regulator of ribosomal protein genes in *C*. *albicans* and other ascomycetes [48, 49] (**S2 Fig**).

Interestingly, 21 of the 66 size mutants identified by our screen have been shown previously to be required for pathogenesis (*p*-value = 1.07e-10; **S1 Fig)**. This set of genes included those with functions in transcriptional control of biofilm and invasive filamentation (*cyr1*, g*cn5*, *ndt80*, *ace2*, *zcf27*) as well as known adhesion genes (*ahr1*, *war1*). This overlap suggested that cell size homeostasis may contribute to *C*. *albicans* fitness inside the host (**Fig 1C** and **S6 Table**).

### Novel Start regulators in *C*. *albicans*

Previous work has shown that disruption of cell growth rate is often accompanied by a small cell size phenotype, for example by mutations in *RP* or *Ribi* genes [23, 50]. To identify *bona fide* negative Start regulators, as opposed to mere growth rate-associated effects, doubling times were determined for the 32 homozygous small size mutants identified in our screens (**S3 Table**). Mutants that exhibited a greater than 10% increase in doubling time as compared to the wt controls were removed from subsequent consideration for this study. As expected, amongst the 21 remaining candidates predicted to more directly couple growth to division (**Table 1**), we recovered two known conserved repressors of Start, namely Sfp1 and Sch9. Candidate Start regulators in *C*. *albicans* included many conserved genes that do not affect size in *S*. *cerevisiae*, including components of the HOG MAPK pathway (Hog1, Pbs2), genes linked to respiration (Hap2, Hap43), invasive filamentous growth (Cph2), adhesion (Ahr1, War1) and metabolism (Ino4, Mig2, Gis2). We also found that inactivation of Nrm1 resulted in *whi* phenotype, consistent with its role as a repressor of the G1/S transition [51]. Interestingly, loss of the transcription factor Hmo1, a main element in the rewired ribosomal gene regulons in *C*. *albicans* [49], caused a small size phenotype. An unexpectedly potent size regulator that emerged from our screens was Dot6, a Myb-like HTH transcription factor that binds to the PAC (Polymerase A and C) motif [52]. The *dot6* deletion was among the smallest mutants identified in our screens. *C*. *albicans* Dot6 is the ortholog of two redundant transcriptional repressors of rRNA and *Ribi* gene expression called Dot6 and Tod6 in *S*. *cerevisiae*, which cause only a minor large size phenotype when deleted together [28].

**Table 1:** Candidate Start regulators in *C*. *albicans*.

Size gene	Orf19 ID	Median size	Size reduction /WT (%)	Doubling time (min)	Function	Homolog in S. cerevisiae
Hap2	orf19.1228	38 ± 1.9	47	132	Transcription factor that regulates low-iron induction of FRP1	YGL237C
Hap43	orf19.681	38 ± 0.9	47	130	Transcription factor required for low iron response	YHL009C
Ahr1	orf19.7381	34 ± 2.1	46	143	Transcription factor that regulates adhesion	
Cph2	orf19.1187	34.1 ± 3.9	46	115	Transcription factor that promotes hyphal growth	YOR032C
Sch9	orf19.829	34 ± 0.2	38	139	Kinase involved in growth control, cell size, filamentation and virulence	YHR205W
Nrm1	orf19.6022	35 ± 0.4	36	139	Transcriptional regulator of cell cycle genes and DNA replication stress	
Gis2	orf19.3182	45 ± 4.3	36	128	Translational activator for mRNAs	YNL255C
Dot6	orf19.2545	49 ± 0.2	32	139	Protein with unknown function	YER088C
Pbs2	orf19.7388	49.2 ± 0.7	31	140	MAPKK with role in osmotic and oxidative stress responses	YJL128C
Hog1	orf19.895	50.2 ± 1.2	30	136	MAP kinase that regulates osmotic and general stress responses	YLR113W
Hmo1	orf19.6645	51 ± 3.1	30	116	Transcription factor that binds upstream of hexose and ergosterol metabolism genes, and cell cycle genes	YDR174W
Asg1	orf19.166	47 ± 2.1	26	103	Gal4 family transcription factor with similarity to *S*. *cerevisiae* Asg1	YIL130W
	orf19.1496	47 ± 1.9	26	120	Putative transcription factor	
Zcf3	orf19.1168	47 ± 1.6	26	118	Putative transcription factor	
Wor2	orf19.5992	47 ± 3.5	26	96	Transcription factor that regulates of white-opaque switching	
Mig2	orf19.5326	47 ± 3.9	26	133	Putative transcription factor	YGL209W
War1	orf19.1035	47 ± 2.3	26	110	Transcription factor with a role in resistance to weak organic acids	YML076C
Ino4	orf19.837.1	47 ± 2.5	26	114	Transcription factor involved in lipid biosynthesis	YOL108C
	orf19.2821	48.4 ± 1.2	24	136	Protein of unknown function	
Sfp1	orf19.5953	57 ± 2.1	20	139	Transcription factor implicated in regulation of RP and *Ribi* genes	YLR403W
Sok1	orf19.451	57.3 ± 1.1	20	141	Protein kinase required for degradation of Nrg1 transcriptional repressor of hyphal growth genes	YDR006C
**WT strains**						
DAY286		71.7 ± 1.9		144		
SN250		64.1 ± 1.5		139		
SFY87		72.1 ± 0.8		138		
CAI4		55 ± 0.9		144		

We demonstrated the effect of six *C*. *albicans* size regulators on the timing of Start by assessing the correlation between size and bud emergence in a synchronous early G1 phase population of cells obtained by centrifugal elutriation. We used this assay to determine the effect of three potent novel size control mutants that conferred a small size phenotype *(ahr1*, *hog1*, *hmo1*) and, as a control, disruption of a conserved known regulator of Start *in S*. *cerevisiae* (*sfp1*). We also characterized two large size mutants, namely *swi4* and a heterozygous deletion of *CLN3*, which is an essential G1 cyclin in *C*. *albicans* [53]. The critical cell size of the four small sized mutants *ahr1*, *hog1*, *hmo1* and *sfp1* was markedly reduced as compared to the wt parental strain (**Fig 1D**), whereas Start was delayed in the *CLN3*/*cln3* and *swi4* strains. These results demonstrate that the transcription factors Ahr1 and Hmo1, and the MAPK Hog1 are novel *bona fide* repressors of Start in *C*. *albicans*, and suggested that aspects of the Start machinery have diverged between *C*. *albicans* and *S*. *cerevisiae*.

### Basal activity of the HOG MAPK pathway delays Start

**Fig 2:**
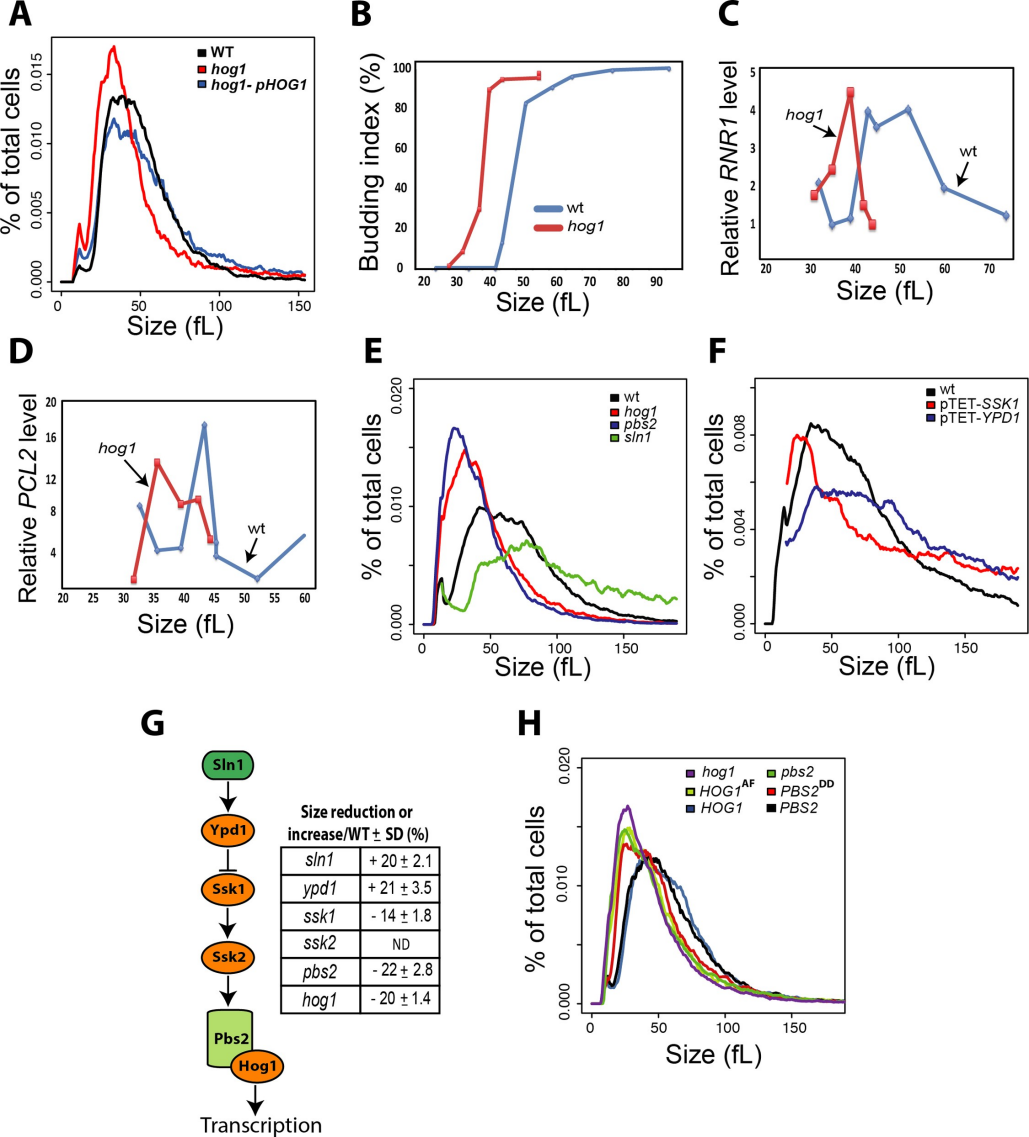
Basal activity of HOG pathway is required for normal Start onset and cell size homeostasis. (**A**) Confirmation of the Whi phenotype in a newly generated *hog1* deletion mutant. Size distributions (i.e., % of cells in each size bin of the Coulter Z2 Channelizer) of a wt (SN148), *hog1* and *hog1* strain complemented with wild type *HOG1* (*hog1*-pHOG1 are shown. (**B-D**) Acceleration of Start in a *hog1* strain. (**B**) Elutriated G1 phase daughter cells were released into fresh media and fractions were collected at intervals of 10 min. Bud emergence was assessed in each size fraction. (**C-D**) Expression of G1/S transcripts. *RNR1* and *PCL2* transcript levels in elutriated cell fractions relative to pre-elutriated asynchronous cells were assessed by quantitative real-time PCR and normalized to *ACT1* levels. (**E-F**) Size distributions of different mutant strains for the HOG pathway in *C*. *albicans*. (**G**) Schematic of the canonical HOG pathway in *C*. *albicans* and summary of size for each mutant strain expressed as mean percentage of reduction or increase of size as compared to the paternal wt strain of each mutant ± standard deviation (four biological replicates). The *ssk2* strain exhibited constitutive filamentation that precluded size determination (ND = not determined). (**H**) Mutation of the two activating phosphorylation sites on Hog1 (T174A and Y176F, termed AF) and Pbs2 (S355D and T359D, termed DD) confers a small size phenotype.

We generated a new *hog1* homozygous deletion mutant in *C*. *albicans* to confirm the small size phenotype (**Fig 2A**). The *hog1* mutant strain had a median cell volume that was 20% smaller than its congenic parental strain, at 44 and 55 fL respectively. To ascertain that this effect was mediated at Start, we evaluated two hallmarks of Start, namely bud emergence and the onset of SBF-dependent transcription as a function of cell size in synchronous G1 phase cells obtained by elutriation. As assessed by median size of cultures in which 25% of cells had a visible bud, the *hog1* mutant passed Start at 41 fL, whereas the parental wt control culture passed Start at 55 fL (**Fig 2B**). Importantly, in the same experiment, the onset of G1/S transcription was accelerated in the *hog1* strain as judged by the peak in expression of the two representative G1 transcripts, *RNR1* and *PCL2* (**Fig 2C and 2D**). These results demonstrated that the Hog1 protein kinase normally acts to delay the onset of Start.

We then tested whether other main elements of the HOG pathway, namely the MAPKK Pbs2, the phosphorelay proteins Ssk1 and Ypd1, and the two-component transducer Sln1, were also required for normal cell size homeostasis (**Fig 2E–2G**). Disruption of the upstream negative regulators (Ypd1 and Sln1) caused a large size whereas mutation of the core MAPK module (Ssk1, Pbs2 and Hog1) caused a small size phenotype. As the cultures for these experiments were grown in constant normo-osmotic conditions, we inferred that the effect of the HOG module on cell size was unrelated to its canonical role in the osmotic stress response. Consistent with this interpretation, mutation of the known osmotic stress effectors of the HOG pathway in *C*. *albicans*, namely the glycerol biosynthetic genes *GPD1*, *GPD2* and *RHR2* [54, 55], did not cause a cell size defect (**S3 Fig**). To address whether basal activity of the HOG MAPK module might be required for size control, we tested the effect of phosphorylation site mutants known to block signal transmission. Mutation of the activating phosphorylation sites on either Hog1 (Thr174 and Tyr176) or Pbs2 (Ser355 and Thr359) to non-phosphorylatable residues phenocopied the small size of *hog1* and *pbs2* deletion mutants, respectively (**Fig 2H**). These results demonstrated that a basal level of Hog1 and Pbs2 activity was required for Start repression under non-stress conditions.

To examine the possible role of the HOG pathway in communicating nutrient status to the Start machinery, the effects of different carbon sources on cell size were assessed in *hog1* and wt strains. Cell size was reduced on poor carbon sources in the *hog1* strain to the same extent as the wt strain, suggesting that the HOG module was not required for carbon source-mediated regulation of cell size (**S4 Fig**). These results demonstrate that the HOG module relays a stress- and carbon source-independent signal for size control to the Start machinery in *C*. *albicans*.

Previous genome-wide screens in *S*. *cerevisiae* failed to uncover a role for the HOG pathway in size control [23, 30–32]. To confirm these results, cell size distributions of HOG pathway mutants in *S*. *cerevisiae* (*hog1*, *pbs2*, *ssk1*, *ssk2*, *opy2* and *sho1* strains) were assessed in rich medium. None of the *S*. *cerevisiae* mutants had any discernable size defect as compared to a parental wt strain (**S5 Fig**).

### Hog1 acts upstream of the SBF transcription factor complex

**Fig 3:**
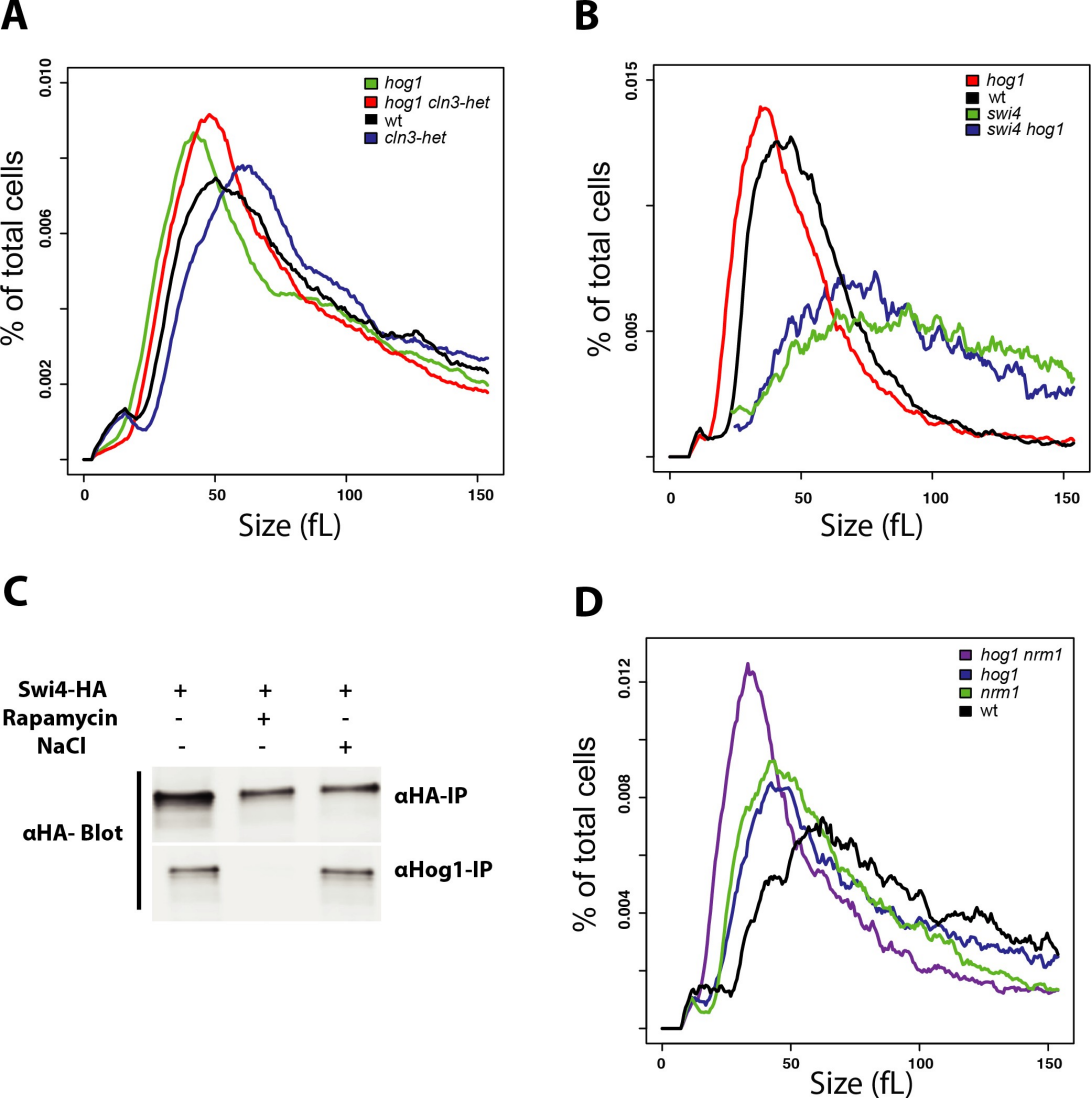
Genetic interactions between the HOG pathway and the G1/S transcriptional machinery. (**A**) Additive effect of *hog1* and *Cln3*/*cln3* mutations on cell size. The wt strain was in the SN148-Arg+ parental background. (**B**) A *swi4* mutation is epistatic to a *hog1* mutation for cell size. The wt strain was in the SN250 background. (**C**) Co-immunoprecipitation assays for Hog1 and Swi4. Cultures were treated or not as indicated with rapamycin (0.5 μg/ml or NaCl (0.5 M) for 30 min. (**D**) Additive effect of *hog1* and *nrm1* mutations on cell size. The wt strain was in the SN250 background.

Cln3-dependent activation of the Swi4-Swi6 transcriptional complex drives G1/S progression in both *S*. *cerevisiae* and *C*. *albicans* [56–59] and we confirmed that *CLN3*/*cln3*, *swi6* and *swi4* mutants all exhibited large size and a G1 phase delay (**Figs 1D and S2E**). To examine the functional relationship between the HOG pathway and these canonical Start regulators, we characterized their genetic interactions by size epistasis. We observed that the small size of a *hog1* mutant strain was partially epistatic to the large size of the heterozygous *CLN3*/*cln3* mutant (**Fig 3A**), suggesting that the HOG pathway may function in parallel to Cln3. In contrast, a *hog1 swi4* double mutant strain had a large size comparable to that of a *swi4* mutant, suggesting that Hog1 acts genetically upstream of Swi4 to inhibit Start (**Fig 3B)**. In support of this finding, co-immunoprecipitation assays revealed that Hog1 physically interacted with Swi4 in a rapamycin-sensitive manner and that the Hog1-Swi4 interaction was insensitive to osmotic stress (**Fig 3C**). In *C*. *albicans*, the Nrm1 inhibitor is known to interact with the SBF complex to repress the G1/S transition [51], and consistently a *nrm1* mutant exhibited a reduced cell size (**Fig 3D**). We found that a *nrm1 hog1* double mutant had a smaller size than either of the *nrm1* or *hog1* single mutants, suggesting that Nrm1 and Hog1 act in parallel pathways to inhibit G1/S transcription (**Fig 3D**). Collectively, these genetic and biochemical results identified Hog1 as a new regulator of SBF in *C*. *albicans*, and suggested that Hog1 may transmit signals from the TOR growth control network to the G1/S machinery.

### The Ptc1 and Ptc2 phosphatases control Start via Hog1

**Fig 4:**
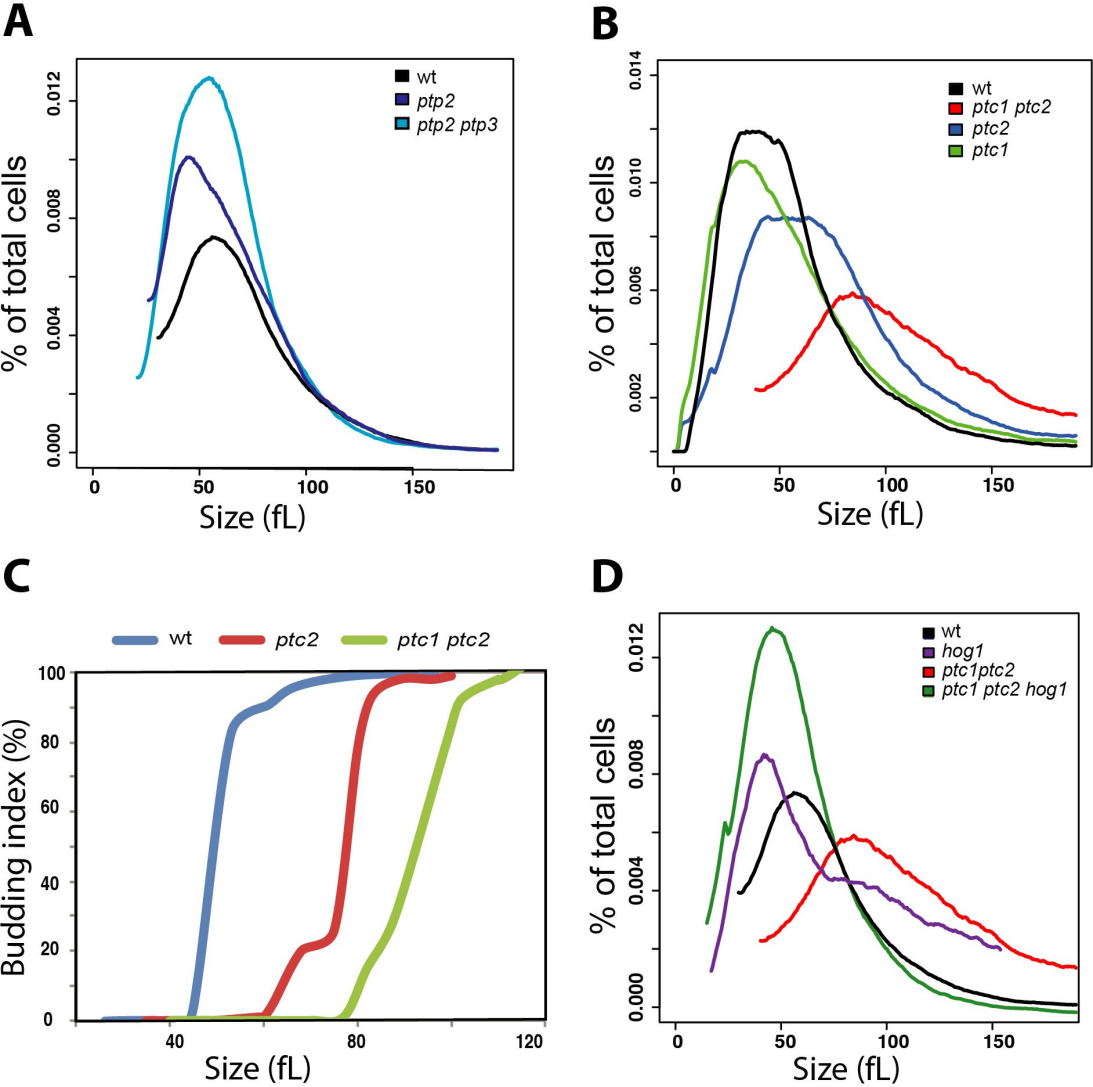
Ptc1 and Ptc2 control Start via Hog1. **(A)** Size distributions of a wt strain and *ptc1*, *ptc2 and ptc1 ptc2* deletion mutants. **(B)** Size distributions of a wt strain, a *ptp2* single mutant, and a *ptp2 ptp3* double mutant. (**C**) Start is delayed in *ptc* mutants. Elutriated G1 phase daughter cells were released into fresh media and monitored for bud emergence as a function of size. (**D**) The small cell size of a *hog1* mutant is epistatic to the large size of a *ptc1 ptc2* double mutant.

MAPK activity is antagonized by the action of serine/threonine (Ser/Thr) phosphatases, tyrosine (Tyr) phosphatases, and dual specificity phosphatases that are able to dephosphorylate both Ser/Thr and Tyr residues [60]. In *S*. *cerevisiae*, after adaptation to osmotic stress, components of the HOG pathway are dephosphorylated by Tyr phosphatases and type 2C Ser/Thr phosphatases [60, 61]. In *C*. *albicans*, recent work has identified the two Tyr phosphatases Ptp2 and Ptp3 as modulators of the basal activity of Hog1 [62]. A prediction of the HOG-dependent size control model is that disruption of the phosphatases that modulate Hog1 basal activity should cause a large cell size. However, none of the Tyr-phosphatase single mutants *ptp1*, *ptp2* or *ptp3*, nor a *ptp2 ptp3* double mutant exhibited a noticeable cell size defect (**Fig 4A**). In contrast, deletion of the type 2C Ser/Thr phosphatase Ptc2 conferred a median size of 84.9 fL, which was 24% larger than the parental wt control size of 68 fL, while a *ptc1 ptc2* double mutant strain had an even larger size of 90.5 fL (**Fig 4B**). To confirm that the large size phenotype of the *ptc* mutants was mediated directly via effects on Start, we evaluated the critical cell size of both *ptc2* and *ptc1 ptc2* mutants in elutriated G1 cells. Whereas wt control cells passed Start at 49 fL, the critical cell size of the *ptc2* and *ptc1 ptc2* mutant strains was increased by 59% to 78 fL and 87% to 92 fL, respectively (**Fig 4C)**. To determine whether Hog1 is an effector of Ptc1 and Ptc2 at Start, we examined the epistatic relationship between the *hog1* and *ptc1 ptc2* mutations. The size of the *hog1 ptc1 ptc2* triple mutant was identical to that of *hog1* single mutant, indicating that Hog1 functions downstream of Ptc1 and Ptc2 for the control of cell size (**Fig 4D**). These data suggest that Ptc1 and Ptc2 phosphatases may modulate the phosphorylation state of Hog1 to govern the timing of Start onset and critical cell size.

### Hog1 activates ribosome biosynthetic gene transcription and inhibits G1/S transcription

**Fig 5:**
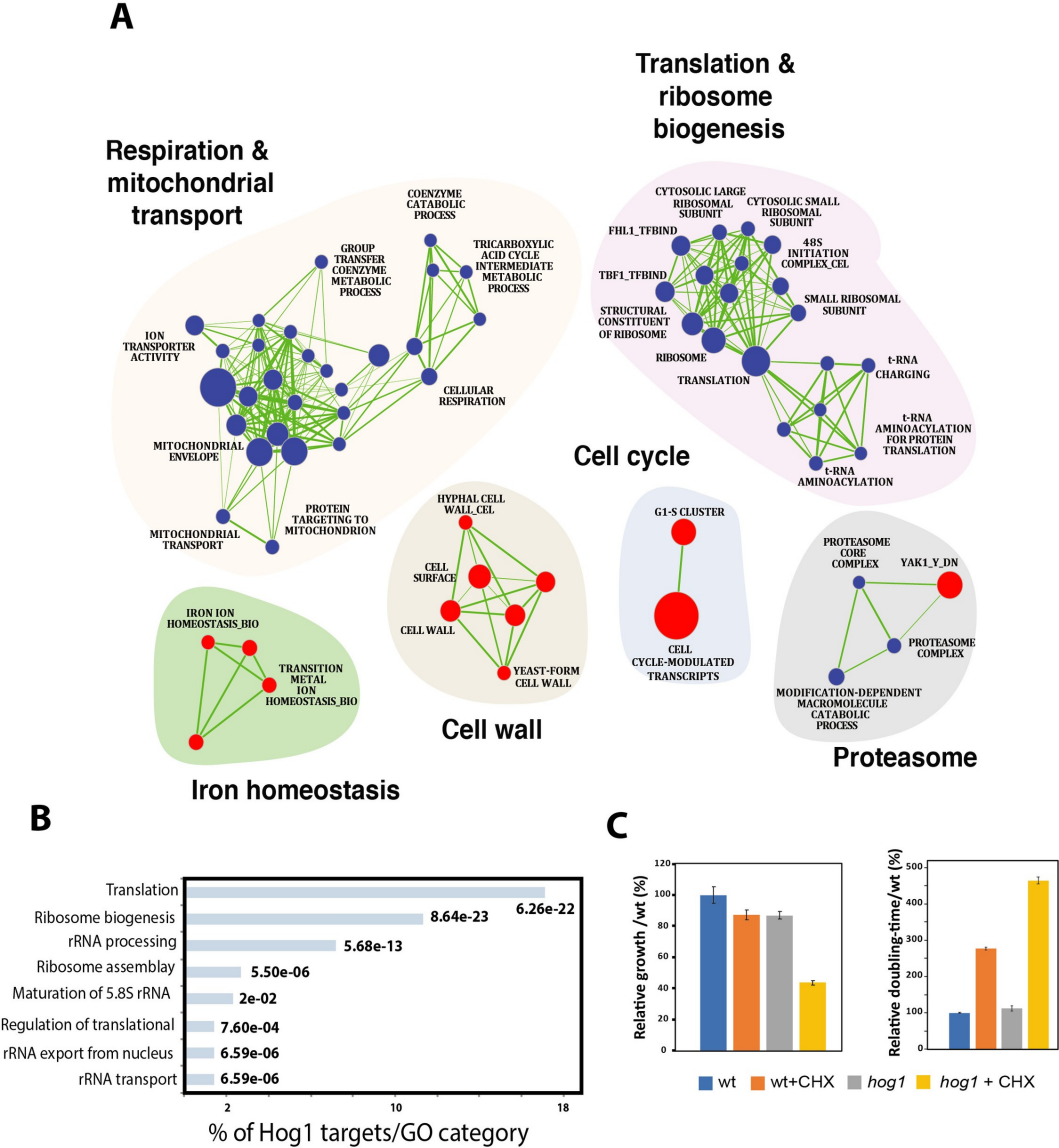
A Hog1-dependent transcriptional program in G1 phase cells. (**A**) GSEA analysis of differentially expressed genes in a *hog1* mutant relative to a congenic wt strain. Cells were synchronized in G1 phase by centrifugal elutriation and released in fresh YPD medium for 15 min and analyzed for gene expression profiles by DNA microarrays. Up-regulated (red circles) and down-regulated (blue circles) transcripts are shown for the indicated processes. The diameter of the circle reflects the number of modulated gene transcripts in each gene set. Known functional connections between related processes are indicated (green lines). Images were generated in Cytoscape with the Enrichment Map plug-in. (**B**) Genome-wide promoter occupancy of Hog1 in G1 phase cells. Gene categories bound by Hog1 were determined by GO term enrichment. *p*-values were calculated using hypergeometric distribution. (**C**) Growth rate and cycloheximide (CHX; 200 μg/ml) sensitivity of wt and *hog1* mutant strains. Relative growth rate was calculated as time to reach half maximal OD_600_ for each culture normalized to the value for the untreated WT control strain, which was 24 h of growth in SC medium at 30°C. Doubling times were calculated during the exponential phase of each strain treated or not with cycloheximide (200 μg/ml) and represented as a percentage relative to the value of the untreated WT control strain. Results are the mean of three replicates. Bars show the means +/- standard errors of the means.

To explore the role of Hog1 at Start, we assessed genome-wide transcriptional profiles using custom microarrays. G1 phase cells for *hog1* mutant and wt strains were collected by centrifugal elutriation, followed by microarray analysis of extracted total RNA. Gene set enrichment analysis (GSEA) of transcriptional profiles [63, 64] revealed that the *hog1* strain was defective in expression of genes that function in protein translation, including members of the 48S/43S translation initiation complex, structural components of the small and large subunits of the ribosome, and tRNA-charging components (**Fig 5A** and **S4 Table**). Transcription of genes that function in mitochondrial transport, the tricarboxylic acid cycle, protein degradation by the 26S proteasome and respiration were also downregulated in a *hog1* deletion. Conversely, the G1/S transcriptional program [56] was hyperactivated in a *hog1* mutant, consistent with the above results for *RNR1* and *PCL2*. These results suggested that Hog1 activates multiple processes that underpin cellular growth in addition to its role as a negative regulator of the G1/S transcriptional program.

It has been previously reported that Hog1 in *S*. *cerevisiae* and its ortholog p38 in humans directly bind and activate downstream transcriptional target genes [65–70]. In *S*. *cerevisiae*, Hog1 thus associates with DNA at stress-responsive genes and is required for recruitment of general transcription factors, chromatin modifying activities and RNA Pol II [66, 69, 71, 72]. However, although mechanisms of Hog1-dependent transcription have been investigated under osmotic stress conditions in *C*. *albicans*, the function of this kinase in normal growth conditions in the absence of stress has not been explored. In order to assess whether Hog1 might directly regulate gene expression relevant to cell size control in *C*. *albicans*, we profiled the genome-wide localization of Hog1 in G1 phase cells obtained by centrifugal elutriation from TAP-tagged Hog1 and untagged control strains. Hog1 binding sites in the genome were determined in duplicate by chromatin immunoprecipitation and microarray analysis (ChIP-chip). These experiments revealed that Hog1^TAP^ was significantly enriched at 276 intergenic regions and 300 ORFs when compared to the untagged control (**S5 Table**). The ORF and promoter targets of Hog1 were strongly represented for translation and *Ribi* genes (**Fig 5B)**, in accord with the above expression profiles. These data suggested that Hog1 may directly activate expression of the *Ribi* regulon and other translation-associated genes. The strong enrichment for Hog1 at translation and *Ribi* loci suggested that Hog1 may be required for maximal translational capacity as G1 phase cells approach Start. Consistently, we observed that a *hog1* mutant exhibited increased sensitivity to the protein translation inhibitor cycloheximide as compared to a wt strain (**Fig 5C**). These results suggest that Hog1 may directly activate ribosome biogenesis and protein translation as cells approach Start.

### Hog1 is required for Sfp1-dependent gene expression and recruitment to target promoters

**Fig 6:**
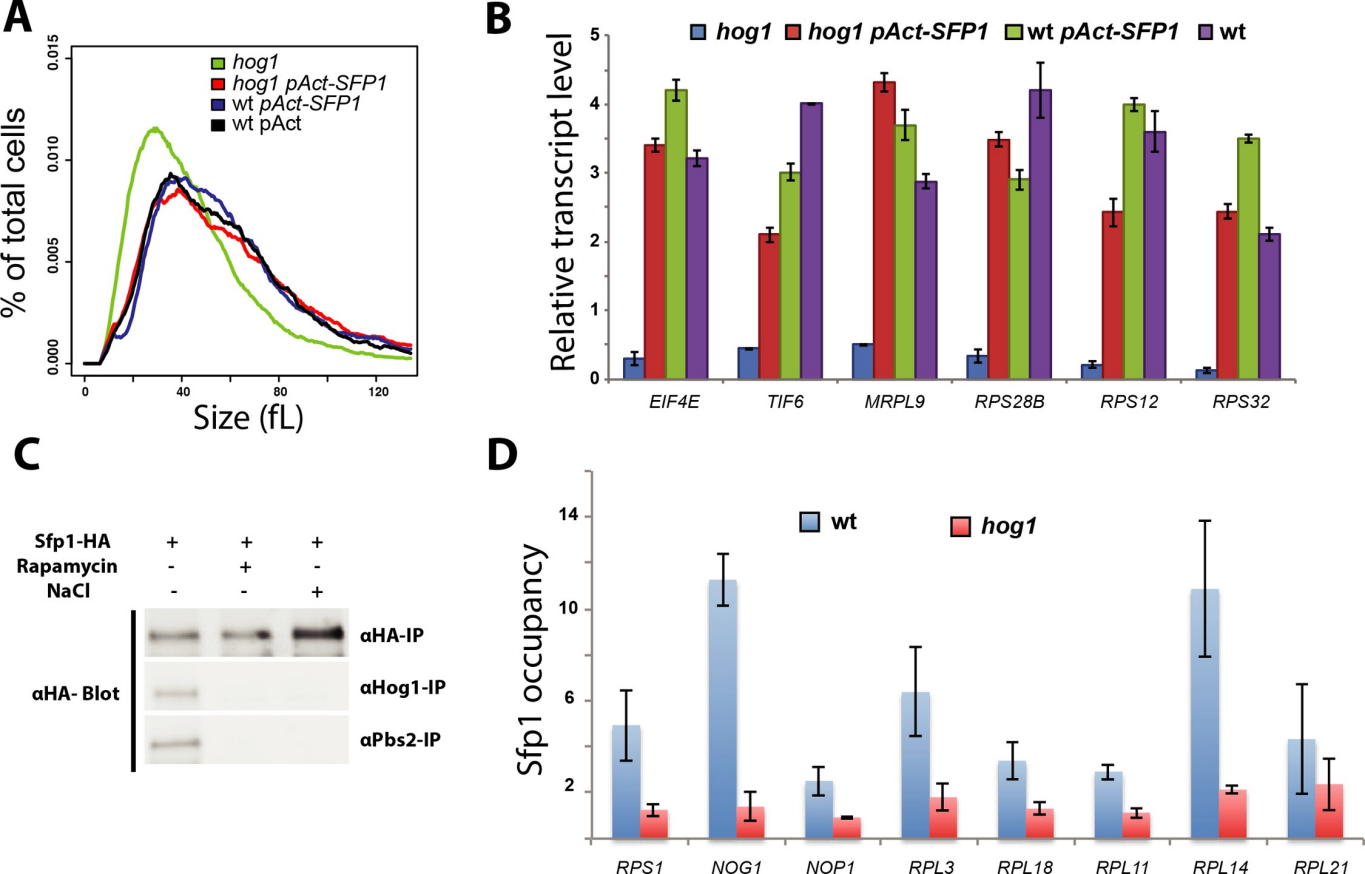
Hog1-dependent recruitment of Sfp1 to promoter DNA. (**A**) Size distributions of wt (wt-pAct), *hog1* (*hog1*/*hog1*), *SFP1*-overexpression (wt/pAct-SFP1) and *hog1 SFP1*-overexpression (*hog1*/*hog1*/pAct-SFP1) strains. (**B**) Increased *SFP1* dosage restores expression of representative *Ribi* and RP transcripts in a *hog1* mutant strain. Relative expression levels of the six transcripts were assessed by real-time qPCR as normalized to *ACT1*. Values are the mean from two independent experiments. (**C**) Sfp1 interactions with Pbs2 and Hog1. Anti-HA immunoprecipitates from a strain bearing an integrated SFP1^HA^ allele grown in the absence or presence of NaCl (0.5 M) or rapamycin (0.5 μg/ml) were probed with anti-HA, anti-Hog1 or anti-Pbs2 antibodies. (**D**) Reduced Sfp1 localization to *Ribi* gene promoters in a *hog1* mutant strain. Values are the mean from three independent ChIP-qPCR experiments for each indicated promoter.

Based on the conserved role of the Sfp1 transcription factor and the kinase Sch9 in ribosome biogenesis and cell size control in *C*. *albicans*, we examined genetic interactions between these factors and the HOG pathway. To identify potential epistatic interactions, we overexpressed *SCH9* or *SFP1* in a *hog1* strain. The overexpression of *SFP1* but not *SCH9* restored the *hog1* strain to a near wt cell size distribution (**Fig 6A**). These results suggested that Sfp1 might act downstream of Hog1. Consistent with this interpretation, we found that the gene expression defects of six *Ribi* and translation genes (*RPS12*, *RPS28B*, *RPS32*, *EIF4E* and *TIF6*) in a *hog1* strain were rescued by the overexpression of *SFP1* (**Fig 6B**).

Given the apparent genetic relationship between Hog1 and Sfp1, we examined whether the two proteins might physically interact. We evaluated the interaction at endogenous levels using a chromosomal HA-tagged Sfp1 allele and polyclonal antibodies that recognize Pbs2 and Hog1. Capture of Sfp1^HA^ from cell lysates followed by antibody detection revealed that Sfp1 interacted with both Pbs2 and Hog1 (**Fig 6C**). Notably, the Sfp1 interaction with both Hog1 and Pbs2 was abolished by either osmotic stress or rapamycin (**Fig 6C**). These results suggested that the timing of Start may be governed in part by modulation of the Hog1-Sfp1 interaction by stress and nutrient signals.

We then examined whether Sfp1 might play an analogous role in Start control in *C*. *albicans* as in *S*. *cerevisiae*. As described above, an *sfp1* deletion strain was extremely small and passed Start at only 42% of wt size (**Figs 1D and S2A**). Consistently, transcriptional profiles of a strain bearing a tetracycline-regulated allele of *SFP1* demonstrated that expression of the *Ribi* regulon was partially Sfp1-dependent (**S6A Fig**). We also found that an *sfp1* strain was as sensitive to the protein translation inhibitor cycloheximide as a *hog1* strain (**S6B Fig**). These data demonstrated that Sfp1 is a transcriptional activator of *Ribi* genes and a negative regulator of Start in *C*. *albicans*.

The finding that both Hog1 and Sfp1 controlled the expression of *Ribi* genes, together with the finding that Hog1 acted upstream of Sfp1, led us to hypothesize that Hog1 might be required for the recruitment of Sfp1 to its target genes. To test this hypothesis, we used ChIP-qPCR to measure *in vivo* promoter occupancy of Sfp1^HA^ at eight representative *Ribi* and RP genes that were also bound by Hog1. While Sfp1 was detected at each of these promoters in a wt strain the ChIP signals were abrogated in the *hog1* mutant strain (**Fig 6D**). From these data, we concluded that Sfp1 regulates the *Ribi* regulon in a Hog1-dependent manner, and that the HOG module lies at the interface of the G1/S transcriptional and growth control machineries in *C*. *albicans*

## Discussion

**Fig 7:**
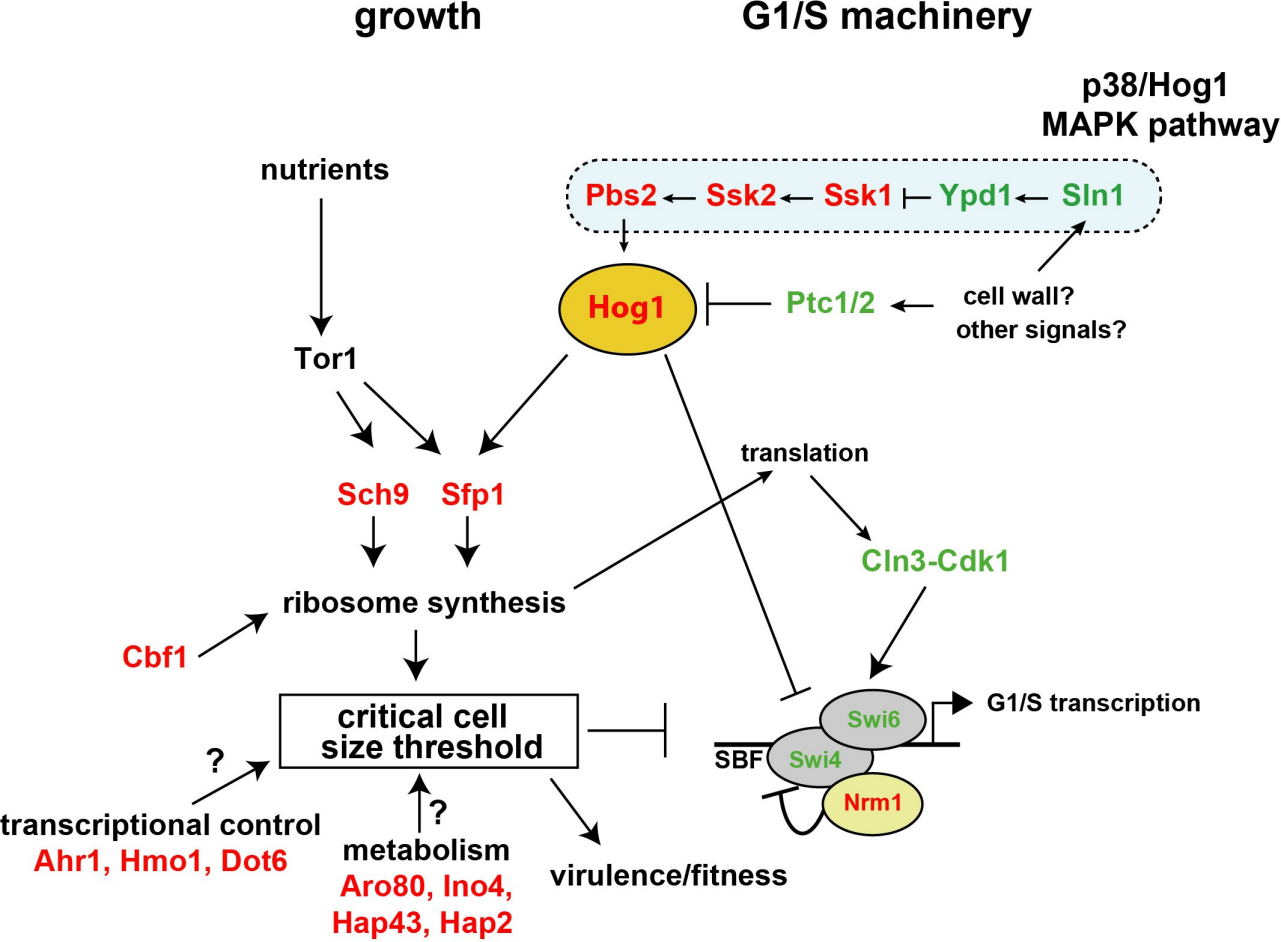
Architecture of the Start machinery in *C*. *albicans*. Hog1 inhibits the SBF G1/S transcription factor complex and in parallel controls Sfp1 occupancy of *Ribi* gene promoters, and thereby directly links growth and division. The activity of Hog1 is modulated by the phosphatases Ptc1 and Ptc2 to govern the timing of Start onset. Parallel Start pathways revealed by genetic interactions with Hog1, as well as other prominent size control genes in *C*. *albicans* revealed by size screens, are also indicated. Other potential size regulators for which gene inactivation led to small and large size phenotypes are indicated in red and green, respectively.

This genetic analysis of size control in *C*. *albicans* represents the first detailed characterization of the mechanisms underlying regulation of growth and division in a pathogenic fungus. As is the case for other species that have been examined to date, cell size in *C*. *albicans* is a complex trait that depends on diverse biological processes and many genes [23, 30–32, 34, 36]. Of particular note, our screen and subsequent molecular genetic analysis uncovered a novel function for Hog1 as a critical nexus of the growth and division machineries. The HOG module thus represents a direct linkage between cell growth and division (**Fig 7)**.

### Conservation and divergence of cell size control mechanisms

Inactivation of genes that control ribosome biogenesis and protein translation in *C*. *albicans* resulted in a small cell size, consistent with the notion that the rate of ribosome biogenesis is a component of the critical size threshold [1, 24]. In particular, mutation of the key conserved *Ribi* regulators Sch9 and Sfp1 dramatically reduced cell size in *C*. *albicans*. Previous studies have shown that several RP and *Ribi* trans-regulatory factors have been evolutionarily rewired in *C*. *albicans* compared to *S*. *cerevisiae* [48]. Consistently, we found that deletion of *CBF1*, which encodes a master transcriptional regulator of *RP* genes in *C*. *albicans* but not *S*. *cerevisiae*, also caused a small size phenotype. Our analysis also unexpectedly revealed that size regulators may switch between positive and negative functions between the two yeasts. For example, mutation of the conserved transcription factor Dot6 that controls rRNA and *Ribi* expression caused a strong Whi phenotype in *C*. *albicans*, in contrast to the Lge phenotype conferred in *S*. *cerevisiae* [28]. These results illustrate the evolutionary plasticity of size control mechanisms at the transcriptional level.

In *C*. *albicans*, the G1/S phase cell cycle machinery remains only partially characterized but nevertheless appears to exhibit disparities compared to *S*. *cerevisiae*. For instance, despite conservation of SBF and Cln3 function [59, 73], the G1/S repressor Whi5 [16, 19] and the G1/S activator Bck2 [74] appear to have been lost in *C*. *albicans*. In *S*. *cerevisiae*, cells lacking *cln3* are viable and able to pass Start due to the redundant role of Bck2 [74], whereas in *C*. *albicans* Cln3 is essential, presumably due to the absence of a Bck2 equivalent [75]. Nrm1 also appears to have replaced Whi5 as it interacts physically with the SBF complex and acts genetically as a repressor of the G1/S transition in *C*. *albicans* [51]. Consistently, we observe that *nrm1* mutant exhibits a reduced cell size as a consequence of accelerated passage through Start. In addition, the promoters of genes that display a peak of expression during the G1/S transition lack the SCB cis-regulatory element recognized by the SBF complex in *S*. *cerevisiae* and are instead enriched in MCB-like motifs [56].

### Control of Start by the HOG network

Our systematic size screen uncovered a new stress-independent role of the HOG signaling network in coordinating cell growth and division. Hog1 and its metazoan counterparts, the p38 MAPK family, respond to various stresses in fungi [76] and metazoans [77]. In contrast to these stress-dependent functions, our data suggests that the basal level activity of the module is required to delay the G1/S transition under non-stressed homeostatic growth conditions. This function of the HOG module appears specific to *C*. *albicans* as compared to *S*. *cerevisiae*. However, the p38 MAPK family is implicated in size control in metazoan species. In the fruit fly *D*. *melanogaster* loss of p38β causes small cell and organism size [78], while in mice inactivation of the two Hog1 paralogs p38γ and p38δ alters both cell and organ size, including in the heart and the liver [79, 80]. Recent elegant work in human cells has shown that p38 MAPK activity enforces size homeostasis by controlling the length of G1 phase in proportion to cell size [81]. In *S*. *pombe*, there are two critical cell size threshold at both G1/S and G2/M phases [1, 5]. Previous studies have shown that the p38/Hog1 homolog in *S*. *pombe*, Sty1, controls mitotic commitment and cell size in a nutrient-dependent manner [34, 82–84]. Deletion of *STY1* resulted in a large cell size phenotype which is in accordance with its role as a positive regulator of mitotic onset [83]. These observations suggest the overall role of Hog1 in size control is conserved and that *C*. *albicans* may be a suitable yeast model to dissect the mechanisms whereby Hog1 links growth to cell cycle commitment. In particular, we show that the entire HOG module is required for cell size control in *C*. *albicans*, and further demonstrate a unique role for the type 2C phosphatases Ptc1 and Ptc2 in size control. In contrast, modulation of the basal activity of Hog1 by the tyrosine phosphatases Ptp2 and Ptp3 in response to a reduction of TOR activity is required for the separate response of hyphal elongation [62]. The mechanisms whereby the same MAPK module can specifically respond to stress, nutrient and cell size remains to be resolved.

A key question raised by our study is the nature of the signal(s) sensed by the HOG network that mediate the coupling of growth to division. Deletion of the upstream negative regulators of the HOG module, Ypd1 and Sln1, caused an increase in cell size, consistent with the negative regulation of Start by the entire HOG network. Previous studies have suggested that Sln1 histidine phosphotransferase activity is required for cell wall biogenesis in both *S*. *cerevisiae* and *C*. *albicans* [85, 86]. Interestingly, we also found that disruption of the beta-1,3-glucan synthase subunit Gsc1 also caused a reduced size in *C*. *albicans* [53]. We speculate that accumulation of cell wall materials, such as glucans, and/or cell wall mechanical proprieties may be sensed through basal activity of the HOG module in order to link growth rate to division. This model is analogous to that postulated in bacteria, whereby the enzymes that synthesize cell wall peptidoglycan help establish cell size control by maintaining cell width [87]. In support of this notion, perturbation of the cell wall leads to a G1 phase cell cycle arrest in *S*. *cerevisiae* via the PKC/Slt2 signalling network [32, 88, 89].

Finally, in addition to its role in size control in *C*. *albicans*, other stress-independent functions have been attributed to the HOG pathway in different fungi. In *Aspergillus fumigatus* and *A*. *nidulans*, Hog1 controls growth [90], conidial germination [91] and sexual development [92, 93]. In *Cryptococcus neoformans*, Hog1 is required for mating and, together with the PKA pathway, contributes to the modulation of cellular response to glucose availability [94, 95]. Future efforts on the mechanisms by which Hog1 control these processes will lend further insights into how this central MAPK conduit transmits multiple different signals.

### The HOG network lies at the nexus of growth and cell cycle control

The nature of the linkage between growth to division represents a longstanding general problem in cell biology. The complex genetics of size control, reflected in the 66 genes identified in this study that directly or indirectly affect size, confounds the notion of a simple model of size control [2]. Our analysis of Hog1 interactions with the known growth and division machineries nevertheless suggests that the HOG module may directly link growth and division to establish the size threshold at Start. We demonstrated that the HOG module acts genetically upstream of Sfp1 to activate *Ribi* and translation-related genes, and specifically that Hog1 is required for the expression of many genes implicated in ribosome biogenesis and the recruitment of Sfp1 to the relevant promoters. We also demonstrated that Hog1 and its upstream kinase Pbs2 both physically interact with Sfp1, and that Hog1 localizes to many ribosome biogenesis promoters, consistent with a direct regulatory mechanism. These data suggest that basal activity of the HOG module help set ribosome biogenesis and protein synthesis rates. The HOG module also exhibits strong genetic interactions with the SBF transcriptional machinery since the loss of SBF function is epistatic to HOG module mutations and Hog1 physically interacts with SBF. The HOG module is therefore ideally positioned to communicate the activity of the growth machinery to the cell cycle machinery. We speculate that under conditions of rapid growth, Hog1 and/or other components of the HOG module may be sequestered away from SBF, thereby delaying the onset of G1/S transcription. In the absence of Hog1 basal activity, this balance is set to a default state, in which SBF is activated prematurely for a given rate of growth. Taken together, these observations suggest a model whereby the HOG module directly links growth to cell cycle commitment (**Fig 7**). The control of SBF by the HOG module appears to operate in parallel to Cln3, Nrm1 and nutrient conditions, suggesting that multiple signals are integrated at the level of SBF, perhaps to optimize adaptation to different conditions [2]. Further analysis of the functional relationships between the HOG module and the numerous other genes that affect size in *C*. *albicans* should provide further insights into the linkage between growth and division.

### Plasticity of the global size control network and organism fitness

It has been argued that optimization of organism size is a dominant evolutionary force because fitness depends exquisitely on adaptation to a particular size niche [96]. The strong link between size and fitness has been elegantly demonstrated through the artificial evolution of *E*. *coli* strains adapted to different growth rates [3]. Comparison of the size phenomes of the opportunistic pathogen *C*. *albicans* and the saprophytic yeasts *S*. *cerevisiae* and *S*. *pombe* reveals many variations in the growth and cell cycle machineries that presumably reflect the different lifestyles of these yeasts. Intriguingly, one third of the size regulators identified in our focused *C*. *albicans* reverse-genetic screens have been previously identified as virulence determinants for this pathogen, similar to our previous study of genes that are haploinsufficient for cell size in *C*. *albicans* [53]. We speculate that cell size may be an important virulence trait. Other fungal pathogens such as *Histoplasma capsulatum*, *Paracoccidioides brasiliensis*, *C*. *neoformans* and *Mucor circinelloides* also exploit cell size as a virulence determinant [97] to access specific niches in the host and/or to escape from host immune cells. In *C*. *albicans*, the recently discovered gray cell type is characterized by a small size, a propensity to cause cutaneous infections, and reduced colonization of internal organs [98, 99]. Conversely, the response of the host immune system appears to sense *C*. *albicans* size to mitigate tissue damage at the site of infection [100]. The evident scope and plasticity of the global size control network provides fertile ground for adaptive mechanisms to optimize organism size and fitness.

## Methods

### Strains, mutant collections and growth conditions

*C*. *albicans* strains were cultured at 30°C in yeast-peptone-dextrose (YPD) medium supplemented with uridine (2% Bacto peptone, 1% yeast extract, 2% w/v dextrose, and 50 mg/ml uridine). Alternative carbon sources (glycerol and ethanol) were used at 2% w/v. Wt and mutant strains used in this study together with diagnostic PCR primers are listed in **S7 Table**. The kinase [41] and the transcriptional factor [42] mutant collections used for cell size screens were acquired from the genetic stock center (http://www.fgsc.net). The transcriptional regulator [45] mutant collection was kindly provided by Dr. Dominique Sanglard (University of Lausanne). Growth assay curves were performed in triplicate in 96-well plate format using a Sunrise plate-reader (Tecan) at 30°C under constant agitation with OD_595_ readings taken every 10 min for 24h. TAP and HA tags were introduced into genomic loci as previously described [101]. Overexpression constructs were generated with the CIp-Act-cyc plasmid which was linearized with the *StuI* restriction enzyme for integrative transformation [102].

### Cell size determination

Cell volume distributions, referred to as cell size, were analyzed on a Z2-Coulter Counter (Beckman). *C*. *albicans* cells were grown overnight in YPD at 30°C, diluted 1000-fold into fresh YPD and grown for 5h at 30°C to an early log phase density of 5x10^6^–10^7^ cells/ml. For the tetracycline repressible mutants, all strains and the wt parental strain CAI-4 were grown overnight in YPD supplemented with the antibiotic doxycycline (40μg/ml) to achieve transcriptional repression. We note that high concentration of doxycycline (100 μg/ml) cause a modest small size phenotype in *C*. *albicans* but the screen concentration of 40 μg/ml doxycycline did not cause an alteration in cell size. 100 μl of log phase (or 10 μl of stationary phase) culture was diluted in 10 ml of Isoton II electrolyte solution, sonicated three times for 10s and the distribution measured at least 3 times in 3 different independent experiments on a Z2-Coulter Counter. Size distributions were normalized to cell counts in each of 256 size bins and size reported as the peak mode value for the distribution. Data analysis and clustering of size distributions were performed using custom R scripts (**S1 File)**.

### Centrifugal elutriation

The critical cell size at Start was determined by plotting budding index as a function of size in synchronous G1 phase fractions obtained using a JE-5.0 elutriation rotor with 40 ml chamber in a J6-Mi centrifuge (Beckman, Fullerton, CA) as described previously [103]. *C*. *albicans* G1 phase cells were released in fresh YPD medium and fractions were harvested at an interval of 10 min to monitor bud index. For the *hog1* mutant strain, additional size fractions were collected to assess transcript levels of the *RNR1*, *PCL2* and *ACT1* as cells progressed through G1 phase at progressively larger sizes.

### Gene expression profiles and quantitative real-time PCR

Overnight cultures of *hog1* mutant and wt strains were diluted to an OD_595_ of 0.1 in 1 L fresh YPD-uridine media, grown at 30°C to an OD_595_ of 0.8 and separated into size fractions by elutriation at 16°C. A total of 10^8^ G1 phase cells were harvested, released into fresh YPD medium and grown for 15 min prior to harvesting by centrifugation and stored at -80°C. Total RNA was extracted using an RNAeasy purification kit (Qiagen) and glass bead lysis in a Biospec Mini 24 bead-beater. Total RNA was eluted, assessed for integrity on an Agilent 2100 Bioanalyzer prior to cDNA labeling, microarray hybridization and analysis [104]. The GSEA Pre-Ranked tool (http://www.broadinstitute.org/gsea/) was used to determine statistical significance of correlations between the transcriptome of the *hog1* mutant with a ranked gene list [105] or GO biological process terms as described by Sellam *et al*. [105]. Data were visualized using the Cytoscape [106] and EnrichmentMap plugin [107]. Gene expression data are available at GEO with the accession number GSE126732. For quantitative real-time PCR (qPCR), cells were grown as for the microarray experiment. cDNA synthesis and qPCR procedure were performed as previously described [108].

### Promoter localization by ChIP-chip and ChIP-qPCR

ChIP analyses were performed as described using a custom Agilent microarray containing 14400 (8300 intergenic and 6100 intragenic) 60-mer oligonucleotides that covered all intergenic regions, ORFs and different categories of non-coding RNAs (tRNAs, snoRNAs, snRNAs and rRNA [101]. A total of 10^7^ G1 phase cells were harvested from log phase cultures by centrifugal elutriation and released into fresh YPD medium for 15 min. Arrays were scanned with a GenePix 4000B Axon scanner, and GenePix Pro software 4.1 was used for quantification of spot intensities and normalization. Hog1 genomic occupancy was determined in duplicate ChIP-chip experiments, which were averaged and thresholded using a cutoff of two standard deviations (SDs) above the mean of log ratios (giving a 2-fold enrichment cutoff). ChIP-chip data are available at GEO with the accession number GSE126732. For ChIP analysis of HA-tagged Sfp1, qPCR was performed using an iQ 96-well PCR system for 40 amplification cycles and QuantiTect SYBR Green PCR master mix (Qiagen) using 1 ng of captured DNA and total genomic DNA extracted from the whole cell extract. The coding sequence of the *C*. *albicans ACT1* gene was used as a reference for background in all experiments. Values were calculated as the mean of triplicate experiments.

### Protein immunoprecipitation and immunoblot

Cultures of epitope-tagged strains were grown to OD_595_ of 1.0–1.5 in YPD and either treated or not with rapamycin (0.2 μg/ml) or NaCl (0.5 M) for 30 min. Cells were harvested by centrifugation and lysed by glass beads in IP150 buffer (50 mM Tris-HCl (pH 7.4), 150 mM NaCl, 2 mM MgCl_2_, 0.1% Nonidet P-40) supplemented with Complete Mini protease inhibitor cocktail tablet (Roche Applied Science) and 1 mM phenylmethylsulfonyl fluoride (PMSF). 1 mg of total protein from clarified lysates was incubated with 50 μl of monoclonal mouse anti-HA (12CA5) antibody (Roche Applied Science), or 20 μl anti-Pbs2 rabbit polyclonal antibody or 20μl anti-Hog1 rabbit polyclonal antibody (Santa Cruz) and captured on 40 μl Protein A-Sepharose beads (GE) at 4°C overnight. Beads were washed three times with IP150 buffer, boiled in SDS-PAGE buffer, and resolved by 4–20% gradient SDS-PAGE. Proteins were transferred onto activated polyvinylidene difluoride (PVDF) membrane and detected by rabbit anti-HA (1:1000) antibody (QED Biosciences) and IRDye680 secondary antibody (LI-COR).

## Supporting information

S1 Fig Biological functions of size control genes in *C*. *albicans*. (**A**) GO biological process term enrichment of all 66 size mutants identified in this study. (**B**) GO term enrichment of small sized mutants. (**C**) GO term enrichment of large sized mutants. *p*-values were calculated based on a hypergeometric distribution (see http://go.princeton.edu/cgi-bin/GOTermFinder).
(TIF)

S2 Fig Size distributions of different *C*. *albicans* size mutants. The indicated mutant strains and congenic wt control strains were grown to early log phase in rich YPD medium and sized on a Beckman Coulter Z2 Channelizer. Size distributions were shown for (**A**) *sfp1*, (**B**) *sch9*, (**C**) *cbf1*, (**D**) *hmo1*, (**E**) *swi4* and *swi6*, (**F**) *pop2* and *ccr4*, (**G**) *ahr1*, and, (**H**) *hog1* and *pbs2* mutants.
(TIF)

S3 Fig Size distributions of mutants defective in glycerol biosynthesis. (**A**) *gpd2* mutant and wt (SN250) strains were grown to early log phase in rich YPD medium prior to cell size determination. (**B**) The indicated wt (CAI4), *gpd1* and *rhr2* strains were grown to early log phase in rich YPD medium in the presence of doxycycline and sized on a Z2 coulter channelizer.
(TIF)

S4 Fig A *hog1* deletion mutant strain adjusts cell size in response to different carbon sources. Size distribution of log-phase cultures of the indicated wt (**A**) and *hog1* (**B**) strains (SN250 background) grown in synthetic glucose (red curve), glycerol (blue) and ethanol (black) medium. Cultures were sized on a Beckman Coulter Z2 Channelizer.
(TIF)

S5 Fig Disruption of central components of *S*. *cerevisiae* HOG network under non-stressed normo-osmotic conditions. Cultures of the indicated strains were grown to early log phase in rich YPD medium and sized on a Beckman Coulter Z2 Channelizer. Wt (BY4741) and *sfp1Δ* strains were included as controls.
(TIF)

S6 Fig Conservation of Sfp1 function in *C*. *albicans* as a transcriptional activator of *Ribi* genes. (**A**) Network visualization of transcriptional changes in a tet-*SFP1*/*sfp1* conditional mutant strain. Genes expressed at reduced (blue) or elevated (red) levels after Sfp1 repression were organized into functionally connected networks (green lines) based on Gene Ontology biological process terms. Node size indicates the magnitude of change. Data were visualized using Cytoscape and the Enrichment Map plug-in. (**B**) A pTET-*SFP1*/*sfp1* conditional mutant exhibited increased sensitivity to the protein translation inhibitor cycloheximide (CHX, 200 μg/ml). Cells were grown in YPD at 30°C, and OD_595_ readings were taken every 10 min on an automated shaker reader.
(TIF)

S1 Table Experimental size data of individual mutant strains from the three different *C*. *albicans* gene mutant collections used in this study. Mean, median and mode size of each strain are indicated.
(XLSX)

S2 Table List of 66 size mutants in *C*. *albicans* that had a greater than 20% increase or decrease in size compared wt control strains. (XLSX)

S3 Table List of 66 smallest and largest mutants in *C*. *albicans* grouped according to GO biological process terms. (XLSX)

S4 Table Gene set enrichment analysis (GSEA) for expression profiles in G1 phase cells determined in a *hog1* strain. (XLSX)

S5 Table Genome-wide promoter occupancy profile of Hog1 in G1 phase cells. (XLSX)

S6 Table Size mutants that exhibit a known virulence defect. Data were extracted from CGD database.
(XLSX)

S7 Table List of *C*. *albicans* strains and primers used in this study. (XLSX)

S1 File Custom R scripts used to analyse the cell size data. (RTF)

## References

[1] Jorgensen P, Tyers M. How cells coordinate growth and division. Curr Biol. 2004;14(23):R1014–27. 10.1016/j.cub.2004.11.027 . 15589139

[2] Kafri M, Metzl-Raz E, Jonas F, Barkai N. Rethinking cell growth models. FEMS Yeast Res. 2016;16(7). 10.1093/femsyr/fow081 . 27650704

[3] Lenski RE, Travisano M. Dynamics of adaptation and diversification: a 10,000-generation experiment with bacterial populations. Proc Natl Acad Sci U S A. 1994;91(15):6808–14. 8041701 10.1073/pnas.91.15.6808 PMC44287

[4] Kafri M, Metzl-Raz E, Jona G, Barkai N. The Cost of Protein Production. Cell Rep. 2016;14(1):22–31. 10.1016/j.celrep.2015.12.015 26725116 PMC4709330

[5] Turner JJ, Ewald JC, Skotheim JM. Cell size control in yeast. Curr Biol. 2012;22(9):R350–9. 10.1016/j.cub.2012.02.041 22575477 PMC3350643

[6] Cook M, Tyers M. Size control goes global. Curr Opin Biotechnol. 2007;18(4):341–50. 10.1016/j.copbio.2007.07.006 . 17768045

[7] Ginzberg MB, Chang N, D'Souza H, Patel N, Kafri R, Kirschner MW. Cell size sensing in animal cells coordinates anabolic growth rates and cell cycle progression to maintain cell size uniformity. Elife. 2018;7 10.7554/eLife.26957 29889021 PMC6031432

[8] Guertin DA, Sabatini DM. Cell Size Control. eLS: John Wiley & Sons, Ltd; 2001.

[9] Zhao ZL, Song N, Huang QY, Liu YP, Zhao HR. [Clinicopathologic features of lung pleomorphic (spindle/giant cell) carcinoma—a report of 17 cases]. Ai Zheng. 2007;26(2):183–8. . 17298750

[10] Russell P, Nurse P. Negative regulation of mitosis by wee1+, a gene encoding a protein kinase homolog. Cell. 1987;49(4):559–67. . 3032459 10.1016/0092-8674(87)90458-2

[11] Sudbery PE, Goodey AR, Carter BL. Genes which control cell proliferation in the yeast Saccharomyces cerevisiae. Nature. 1980;288(5789):401–4. . 7001255 10.1038/288401a0

[12] Nurse P. Genetic control of cell size at cell division in yeast. Nature. 1975;256(5518):547–51. Epub 1975/08/14. . 1165770 10.1038/256547a0

[13] Nash R, Tokiwa G, Anand S, Erickson K, Futcher AB. The WHI1+ gene of Saccharomyces cerevisiae tethers cell division to cell size and is a cyclin homolog. EMBO J. 1988;7(13):4335–46. 2907481 10.1002/j.1460-2075.1988.tb03332.x PMC455150

[14] Cross FR. DAF1, a mutant gene affecting size control, pheromone arrest, and cell cycle kinetics of Saccharomyces cerevisiae. Mol Cell Biol. 1988;8(11):4675–84. 3062366 10.1128/mcb.8.11.4675-4684.1988 PMC365557

[15] Tyers M. Cell cycle goes global. Curr Opin Cell Biol. 2004;16(6):602–13. 10.1016/j.ceb.2004.09.013 . 15530770

[16] Costanzo M, Nishikawa JL, Tang X, Millman JS, Schub O, Breitkreuz K, et al CDK activity antagonizes Whi5, an inhibitor of G1/S transcription in yeast. Cell. 2004;117(7):899–913. 10.1016/j.cell.2004.05.024 . 15210111

[17] de Bruin RA, Kalashnikova TI, Chahwan C, McDonald WH, Wohlschlegel J, Yates J 3rd, et al Constraining G1-specific transcription to late G1 phase: the MBF-associated corepressor Nrm1 acts via negative feedback. Mol Cell. 2006;23(4):483–96. 10.1016/j.molcel.2006.06.025 . 16916637

[18] Travesa A, Kalashnikova TI, de Bruin RA, Cass SR, Chahwan C, Lee DE, et al Repression of G1/S transcription is mediated via interaction of the GTB motifs of Nrm1 and Whi5 with Swi6. Mol Cell Biol. 2013;33(8):1476–86. 10.1128/MCB.01333-12 23382076 PMC3624247

[19] de Bruin RA, McDonald WH, Kalashnikova TI, Yates J 3rd, Wittenberg C. Cln3 activates G1-specific transcription via phosphorylation of the SBF bound repressor Whi5. Cell. 2004;117(7):887–98. 10.1016/j.cell.2004.05.025 . 15210110

[20] Schaefer JB, Breeden LL. RB from a bud's eye view. Cell. 2004;117(7):849–50. 10.1016/j.cell.2004.06.008 . 15210104

[21] Ginzberg MB, Kafri R, Kirschner M. Cell biology. On being the right (cell) size. Science. 2015;348(6236):1245075 10.1126/science.1245075 25977557 PMC4533982

[22] Schmoller KM, Skotheim JM. The Biosynthetic Basis of Cell Size Control. Trends Cell Biol. 2015;25(12):793–802. Epub 2015/11/18. 10.1016/j.tcb.2015.10.006 . 26573465 PMC6773270

[23] Jorgensen P, Nishikawa JL, Breitkreutz BJ, Tyers M. Systematic identification of pathways that couple cell growth and division in yeast. Science. 2002;297(5580):395–400. 10.1126/science.1070850 . 12089449

[24] Jorgensen P, Rupes I, Sharom JR, Schneper L, Broach JR, Tyers M. A dynamic transcriptional network communicates growth potential to ribosome synthesis and critical cell size. Genes Dev. 2004;18(20):2491–505. 10.1101/gad.1228804 15466158 PMC529537

[25] Lempiainen H, Uotila A, Urban J, Dohnal I, Ammerer G, Loewith R, et al Sfp1 interaction with TORC1 and Mrs6 reveals feedback regulation on TOR signaling. Mol Cell. 2009;33(6):704–16. 10.1016/j.molcel.2009.01.034 . 19328065

[26] Marion RM, Regev A, Segal E, Barash Y, Koller D, Friedman N, et al Sfp1 is a stress- and nutrient-sensitive regulator of ribosomal protein gene expression. Proc Natl Acad Sci U S A. 2004;101(40):14315–22. 10.1073/pnas.0405353101 15353587 PMC521938

[27] Singh J, Tyers M. A Rab escort protein integrates the secretion system with TOR signaling and ribosome biogenesis. Genes Dev. 2009;23(16):1944–58. 10.1101/gad.1804409 19684114 PMC2725937

[28] Huber A, French SL, Tekotte H, Yerlikaya S, Stahl M, Perepelkina MP, et al Sch9 regulates ribosome biogenesis via Stb3, Dot6 and Tod6 and the histone deacetylase complex RPD3L. EMBO J. 2011;30(15):3052–64. 10.1038/emboj.2011.221 21730963 PMC3160192

[29] Davie E, Petersen J. Environmental control of cell size at division. Curr Opin Cell Biol. 2012;24(6):838–44. 10.1016/j.ceb.2012.08.003 . 22947494

[30] Zhang J, Schneider C, Ottmers L, Rodriguez R, Day A, Markwardt J, et al Genomic scale mutant hunt identifies cell size homeostasis genes in S. cerevisiae. Curr Biol. 2002;12(23):1992–2001. . 12477387 10.1016/s0960-9822(02)01305-2

[31] Dungrawala H, Hua H, Wright J, Abraham L, Kasemsri T, McDowell A, et al Identification of new cell size control genes in S. cerevisiae. Cell Div. 2012;7(1):24 10.1186/1747-1028-7-24 23234503 PMC3541103

[32] Soifer I, Barkai N. Systematic identification of cell size regulators in budding yeast. Mol Syst Biol. 2014;10:761 10.15252/msb.20145345 25411401 PMC4299602

[33] Ferrezuelo F, Colomina N, Palmisano A, Gari E, Gallego C, Csikasz-Nagy A, et al The critical size is set at a single-cell level by growth rate to attain homeostasis and adaptation. Nat Commun. 2012;3:1012 10.1038/ncomms2015 . 22910358

[34] Navarro FJ, Nurse P. A systematic screen reveals new elements acting at the G2/M cell cycle control. Genome Biol. 2012;13(5):R36 10.1186/gb-2012-13-5-r36 22624651 PMC3446289

[35] Moris N, Shrivastava J, Jeffery L, Li JJ, Hayles J, Nurse P. A genome-wide screen to identify genes controlling the rate of entry into mitosis in fission yeast. Cell Cycle. 2016;15(22):3121–30. 10.1080/15384101.2016.1242535 27736299 PMC5134717

[36] Bjorklund M, Taipale M, Varjosalo M, Saharinen J, Lahdenpera J, Taipale J. Identification of pathways regulating cell size and cell-cycle progression by RNAi. Nature. 2006;439(7079):1009–13. 10.1038/nature04469 . 16496002

[37] Berman J, Sudbery PE. Candida Albicans: a molecular revolution built on lessons from budding yeast. Nat Rev Genet. 2002;3(12):918–30. 10.1038/nrg948 . 12459722

[38] Odds FC. Candida infections: an overview. Crit Rev Microbiol. 1987;15(1):1–5. 10.3109/10408418709104444 . 3319417

[39] Lavoie H, Hogues H, Whiteway M. Rearrangements of the transcriptional regulatory networks of metabolic pathways in fungi. Curr Opin Microbiol. 2009;12(6):655–63. 10.1016/j.mib.2009.09.015 19875326 PMC3838361

[40] Sellam A, Askew C, Epp E, Lavoie H, Whiteway M, Nantel A. Genome-wide mapping of the coactivator Ada2p yields insight into the functional roles of SAGA/ADA complex in Candida albicans. Mol Biol Cell. 2009;20(9):2389–400. Epub 2009/03/13. 10.1091/mbc.E08-11-1093 19279142 PMC2675619

[41] Blankenship JR, Fanning S, Hamaker JJ, Mitchell AP. An extensive circuitry for cell wall regulation in Candida albicans. PLoS Pathog. 2010;6(2):e1000752 Epub 2010/02/09. 10.1371/journal.ppat.1000752 . 20140194 PMC2816693

[42] Homann OR, Dea J, Noble SM, Johnson AD. A phenotypic profile of the Candida albicans regulatory network. PLoS Genet. 2009;5(12):e1000783 Epub 2009/12/31. 10.1371/journal.pgen.1000783 . 20041210 PMC2790342

[43] Sandai D, Yin Z, Selway L, Stead D, Walker J, Leach MD, et al The evolutionary rewiring of ubiquitination targets has reprogrammed the regulation of carbon assimilation in the pathogenic yeast Candida albicans. MBio. 2012;3(6). 10.1128/mBio.00495-12 23232717 PMC3520108

[44] Noble SM, Johnson AD. Genetics of Candida albicans, a diploid human fungal pathogen. Annu Rev Genet. 2007;41:193–211. 10.1146/annurev.genet.41.042007.170146 . 17614788

[45] Vandeputte P, Pradervand S, Ischer F, Coste AT, Ferrari S, Harshman K, et al Identification and functional characterization of Rca1, a transcription factor involved in both antifungal susceptibility and host response in Candida albicans. Eukaryot Cell. 2012;11(7):916–31. Epub 2012/05/15. 10.1128/EC.00134-12 22581526 PMC3416494

[46] Sudbery P. Cell biology. When wee meets whi. Science. 2002;297(5580):351–2. 10.1126/science.1073042 . 12130772

[47] Noble SM, French S, Kohn LA, Chen V, Johnson AD. Systematic screens of a Candida albicans homozygous deletion library decouple morphogenetic switching and pathogenicity. Nat Genet. 2010;42(7):590–8. Epub 2010/06/15. 10.1038/ng.605 20543849 PMC2893244

[48] Hogues H, Lavoie H, Sellam A, Mangos M, Roemer T, Purisima E, et al Transcription factor substitution during the evolution of fungal ribosome regulation. Mol Cell. 2008;29(5):552–62. Epub 2008/03/18. 10.1016/j.molcel.2008.02.006 18342603 PMC3838363

[49] Lavoie H, Hogues H, Mallick J, Sellam A, Nantel A, Whiteway M. Evolutionary tinkering with conserved components of a transcriptional regulatory network. PLoS Biol. 2010;8(3):e1000329 10.1371/journal.pbio.1000329 20231876 PMC2834713

[50] Tyson CB, Lord PG, Wheals AE. Dependency of size of Saccharomyces cerevisiae cells on growth rate. J Bacteriol. 1979;138(1):92–8. 374379 10.1128/jb.138.1.92-98.1979 PMC218242

[51] Ofir A, Hofmann K, Weindling E, Gildor T, Barker KS, Rogers PD, et al Role of a Candida albicans Nrm1/Whi5 homologue in cell cycle gene expression and DNA replication stress response. Mol Microbiol. 2012;84(4):778–94. 10.1111/j.1365-2958.2012.08056.x 22463761 PMC3345080

[52] Zhu C, Byers KJ, McCord RP, Shi Z, Berger MF, Newburger DE, et al High-resolution DNA-binding specificity analysis of yeast transcription factors. Genome Res. 2009;19(4):556–66. Epub 2009/01/23. 10.1101/gr.090233.108 19158363 PMC2665775

[53] Chaillot J, Cook MA, Corbeil J, Sellam A. Genome-Wide Screen for Haploinsufficient Cell Size Genes in the Opportunistic Yeast Candida albicans. G3 (Bethesda). 2017;7(2):355–60. Epub 2017/01/04. 10.1534/g3.116.037986 28040776 PMC5295585

[54] Kayingo G, Wong B. The MAP kinase Hog1p differentially regulates stress-induced production and accumulation of glycerol and D-arabitol in Candida albicans. Microbiology. 2005;151(Pt 9):2987–99. 10.1099/mic.0.28040-0 . 16151209

[55] San Jose C, Monge RA, Perez-Diaz R, Pla J, Nombela C. The mitogen-activated protein kinase homolog HOG1 gene controls glycerol accumulation in the pathogenic fungus Candida albicans. J Bacteriol. 1996;178(19):5850–2. 8824643 10.1128/jb.178.19.5850-5852.1996 PMC178437

[56] Cote P, Hogues H, Whiteway M. Transcriptional analysis of the Candida albicans cell cycle. Mol Biol Cell. 2009;20(14):3363–73. 10.1091/mbc.E09-03-0210 19477921 PMC2710843

[57] Hussein B, Huang H, Glory A, Osmani A, Kaminskyj S, Nantel A, et al G1/S transcription factor orthologues Swi4p and Swi6p are important but not essential for cell proliferation and influence hyphal development in the fungal pathogen Candida albicans. Eukaryot Cell. 2011;10(3):384–97. 10.1128/EC.00278-10 21257795 PMC3067467

[58] Wittenberg C, Reed SI. Cell cycle-dependent transcription in yeast: promoters, transcription factors, and transcriptomes. Oncogene. 2005;24(17):2746–55. 10.1038/sj.onc.1208606 . 15838511

[59] Bachewich C, Whiteway M. Cyclin Cln3p links G1 progression to hyphal and pseudohyphal development in Candida albicans. Eukaryot Cell. 2005;4(1):95–102. 10.1128/EC.4.1.95-102.2005 15643065 PMC544164

[60] Saito H, Tatebayashi K. Regulation of the osmoregulatory HOG MAPK cascade in yeast. J Biochem. 2004;136(3):267–72. 10.1093/jb/mvh135 . 15598881

[61] Brewster JL, Gustin MC. Hog1: 20 years of discovery and impact. Sci Signal. 2014;7(343):re7 10.1126/scisignal.2005458 . 25227612

[62] Su C, Lu Y, Liu H. Reduced TOR signaling sustains hyphal development in Candida albicans by lowering Hog1 basal activity. Mol Biol Cell. 2013;24(3):385–97. 10.1091/mbc.E12-06-0477 23171549 PMC3564525

[63] Subramanian A, Tamayo P, Mootha VK, Mukherjee S, Ebert BL, Gillette MA, et al Gene set enrichment analysis: a knowledge-based approach for interpreting genome-wide expression profiles. Proc Natl Acad Sci U S A. 2005;102(43):15545–50. 10.1073/pnas.0506580102 16199517 PMC1239896

[64] Sellam A, Tebbji F, Whiteway M, Nantel A. A novel role for the transcription factor Cwt1p as a negative regulator of nitrosative stress in Candida albicans. PLoS One. 2012;7(8):e43956 10.1371/journal.pone.0043956 22952822 PMC3430608

[65] Proft M, Mas G, de Nadal E, Vendrell A, Noriega N, Struhl K, et al The stress-activated Hog1 kinase is a selective transcriptional elongation factor for genes responding to osmotic stress. Mol Cell. 2006;23(2):241–50. 10.1016/j.molcel.2006.05.031 . 16857590

[66] Proft M, Struhl K. Hog1 kinase converts the Sko1-Cyc8-Tup1 repressor complex into an activator that recruits SAGA and SWI/SNF in response to osmotic stress. Mol Cell. 2002;9(6):1307–17. . 12086627 10.1016/s1097-2765(02)00557-9

[67] Pokholok DK, Zeitlinger J, Hannett NM, Reynolds DB, Young RA. Activated signal transduction kinases frequently occupy target genes. Science. 2006;313(5786):533–6. 10.1126/science.1127677 . 16873666

[68] Alepuz PM, Jovanovic A, Reiser V, Ammerer G. Stress-induced map kinase Hog1 is part of transcription activation complexes. Mol Cell. 2001;7(4):767–77. . 11336700 10.1016/s1097-2765(01)00221-0

[69] Alepuz PM, de Nadal E, Zapater M, Ammerer G, Posas F. Osmostress-induced transcription by Hot1 depends on a Hog1-mediated recruitment of the RNA Pol II. EMBO J. 2003;22(10):2433–42. 10.1093/emboj/cdg243 12743037 PMC156001

[70] Simone C, Forcales SV, Hill DA, Imbalzano AN, Latella L, Puri PL. p38 pathway targets SWI-SNF chromatin-remodeling complex to muscle-specific loci. Nat Genet. 2004;36(7):738–43. 10.1038/ng1378 . 15208625

[71] Zapater M, Sohrmann M, Peter M, Posas F, de Nadal E. Selective requirement for SAGA in Hog1-mediated gene expression depending on the severity of the external osmostress conditions. Mol Cell Biol. 2007;27(11):3900–10. 10.1128/MCB.00089-07 17403898 PMC1900016

[72] Nadal-Ribelles M, Conde N, Flores O, Gonzalez-Vallinas J, Eyras E, Orozco M, et al Hog1 bypasses stress-mediated down-regulation of transcription by RNA polymerase II redistribution and chromatin remodeling. Genome Biol. 2012;13(11):R106 10.1186/gb-2012-13-11-r106 23158682 PMC3580498

[73] Chapa y Lazo B, Bates S, Sudbery P. The G1 cyclin Cln3 regulates morphogenesis in Candida albicans. Eukaryot Cell. 2005;4(1):90–4. 10.1128/EC.4.1.90-94.2005 15643064 PMC544163

[74] Wijnen H, Futcher B. Genetic analysis of the shared role of CLN3 and BCK2 at the G(1)-S transition in Saccharomyces cerevisiae. Genetics. 1999;153(3):1131–43. 10545447 10.1093/genetics/153.3.1131 PMC1460821

[75] O'Meara TR, Veri AO, Ketela T, Jiang B, Roemer T, Cowen LE. Global analysis of fungal morphology exposes mechanisms of host cell escape. Nat Commun. 2015;6:6741 10.1038/ncomms7741 25824284 PMC4382923

[76] Krantz M, Becit E, Hohmann S. Comparative genomics of the HOG-signalling system in fungi. Curr Genet. 2006;49(3):137–51. 10.1007/s00294-005-0038-x . 16468042

[77] Saito H, Posas F. Response to hyperosmotic stress. Genetics. 2012;192(2):289–318. 10.1534/genetics.112.140863 23028184 PMC3454867

[78] Cully M, Genevet A, Warne P, Treins C, Liu T, Bastien J, et al A role for p38 stress-activated protein kinase in regulation of cell growth via TORC1. Mol Cell Biol. 2010;30(2):481–95. 10.1128/MCB.00688-09 19917724 PMC2798466

[79] Gonzalez-Teran B, Lopez JA, Rodriguez E, Leiva L, Martinez-Martinez S, Bernal JA, et al p38gamma and delta promote heart hypertrophy by targeting the mTOR-inhibitory protein DEPTOR for degradation. Nat Commun. 2016;7:10477 10.1038/ncomms10477 . 26795633 PMC5476828

[80] Tormos AM, Arduini A, Talens-Visconti R, del Barco Barrantes I, Nebreda AR, Sastre J. Liver-specific p38alpha deficiency causes reduced cell growth and cytokinesis failure during chronic biliary cirrhosis in mice. Hepatology. 2013;57(5):1950–61. 10.1002/hep.26174 . 23354775

[81] Liu S, Ginzberg MB, Patel N, Hild M, Leung B, Li Z, et al Size uniformity of animal cells is actively maintained by a p38 MAPK-dependent regulation of G1-length. Elife. 2018;7 10.7554/eLife.26947 29595474 PMC5876018

[82] Shiozaki K, Russell P. Cell-cycle control linked to extracellular environment by MAP kinase pathway in fission yeast. Nature. 1995;378(6558):739–43. 10.1038/378739a0 . 7501024

[83] Petersen J, Nurse P. TOR signalling regulates mitotic commitment through the stress MAP kinase pathway and the Polo and Cdc2 kinases. Nat Cell Biol. 2007;9(11):1263–72. 10.1038/ncb1646 . 17952063

[84] Millar JB, Buck V, Wilkinson MG. Pyp1 and Pyp2 PTPases dephosphorylate an osmosensing MAP kinase controlling cell size at division in fission yeast. Genes Dev. 1995;9(17):2117–30. . 7657164 10.1101/gad.9.17.2117

[85] Kruppa M, Jabra-Rizk MA, Meiller TF, Calderone R. The histidine kinases of Candida albicans: regulation of cell wall mannan biosynthesis. FEMS Yeast Res. 2004;4(4–5):409–16. 10.1016/S1567-1356(03)00201-0 . 14734021

[86] Rauceo JM, Blankenship JR, Fanning S, Hamaker JJ, Deneault JS, Smith FJ, et al Regulation of the Candida albicans cell wall damage response by transcription factor Sko1 and PAS kinase Psk1. Mol Biol Cell. 2008;19(7):2741–51. 10.1091/mbc.E08-02-0191 18434592 PMC2441657

[87] Chien AC, Hill NS, Levin PA. Cell size control in bacteria. Current Biology. 2012;22(9):R340–9. 10.1016/j.cub.2012.02.032 22575476 PMC3350639

[88] Kono K, Al-Zain A, Schroeder L, Nakanishi M, Ikui AE. Plasma membrane/cell wall perturbation activates a novel cell cycle checkpoint during G1 in Saccharomyces cerevisiae. Proc Natl Acad Sci U S A. 2016;113(25):6910–5. 10.1073/pnas.1523824113 27274080 PMC4922162

[89] Levin DE. Regulation of cell wall biogenesis in Saccharomyces cerevisiae: the cell wall integrity signaling pathway. Genetics. 2011;189(4):1145–75. 10.1534/genetics.111.128264 22174182 PMC3241422

[90] Du C, Sarfati J, Latge JP, Calderone R. The role of the sakA (Hog1) and tcsB (sln1) genes in the oxidant adaptation of Aspergillus fumigatus. Med Mycol. 2006;44(3):211–8. Epub 2006/05/17. 10.1080/13693780500338886 . 16702099

[91] Xue T, Nguyen CK, Romans A, May GS. A mitogen-activated protein kinase that senses nitrogen regulates conidial germination and growth in Aspergillus fumigatus. Eukaryot Cell. 2004;3(2):557–60. Epub 2004/04/13. 10.1128/EC.3.2.557-560.2004 15075285 PMC387654

[92] Kawasaki L, Sanchez O, Shiozaki K, Aguirre J. SakA MAP kinase is involved in stress signal transduction, sexual development and spore viability in Aspergillus nidulans. Mol Microbiol. 2002;45(4):1153–63. Epub 2002/08/16. . 12180932 10.1046/j.1365-2958.2002.03087.x

[93] Jaimes-Arroyo R, Lara-Rojas F, Bayram O, Valerius O, Braus GH, Aguirre J. The SrkA Kinase Is Part of the SakA Mitogen-Activated Protein Kinase Interactome and Regulates Stress Responses and Development in Aspergillus nidulans. Eukaryot Cell. 2015;14(5):495–510. Epub 2015/03/31. 10.1128/EC.00277-14 25820520 PMC4421008

[94] Bahn YS, Kojima K, Cox GM, Heitman J. Specialization of the HOG pathway and its impact on differentiation and virulence of Cryptococcus neoformans. Mol Biol Cell. 2005;16(5):2285–300. Epub 2005/02/25. 10.1091/mbc.E04-11-0987 15728721 PMC1087235

[95] Banerjee D, Bloom AL, Panepinto JC. Opposing PKA and Hog1 signals control the post-transcriptional response to glucose availability in Cryptococcus neoformans. Mol Microbiol. 2016;102(2):306–20. Epub 2016/07/09. 10.1111/mmi.13461 27387858 PMC5055444

[96] Bonner J. Why Size Matters: From Bacteria to Blue Whales. Princeton University Press ed2006.

[97] Wang L, Lin X. Morphogenesis in fungal pathogenicity: shape, size, and surface. PLoS Pathog. 2012;8(12):e1003027 Epub 2012/12/14. 10.1371/journal.ppat.1003027 23236274 PMC3516537

[98] Tao L, Du H, Guan G, Dai Y, Nobile CJ, Liang W, et al Discovery of a "white-gray-opaque" tristable phenotypic switching system in candida albicans: roles of non-genetic diversity in host adaptation. PLoS Biol. 2014;12(4):e1001830 10.1371/journal.pbio.1001830 24691005 PMC3972085

[99] Sellam A, Whiteway M. Recent advances on Candida albicans biology and virulence [version 1; referees: 2 approved]. 2016;5(2582). 10.12688/f1000research.9617.1. 27853524 PMC5089126

[100] Branzk N, Lubojemska A, Hardison SE, Wang Q, Gutierrez MG, Brown GD, et al Neutrophils sense microbe size and selectively release neutrophil extracellular traps in response to large pathogens. Nat Immunol. 2014;15(11):1017–25. 10.1038/ni.2987 25217981 PMC4236687

[101] Lavoie H, Sellam A, Askew C, Nantel A, Whiteway M. A toolbox for epitope-tagging and genome-wide location analysis in Candida albicans. BMC Genomics. 2008;9:578 Epub 2008/12/06. 10.1186/1471-2164-9-578 19055720 PMC2607300

[102] Blackwell C, Russell CL, Argimon S, Brown AJ, Brown JD. Protein A-tagging for purification of native macromolecular complexes from Candida albicans. Yeast. 2003;20(15):1235–41. 10.1002/yea.1036 . 14618561

[103] Tyers M, Tokiwa G, Futcher B. Comparison of the Saccharomyces cerevisiae G1 cyclins: Cln3 may be an upstream activator of Cln1, Cln2 and other cyclins. EMBO J. 1993;12(5):1955–68. 8387915 10.1002/j.1460-2075.1993.tb05845.x PMC413417

[104] Sellam A, Tebbji F, Nantel A. Role of Ndt80p in sterol metabolism regulation and azole resistance in Candida albicans. Eukaryot Cell. 2009;8(8):1174–83. Epub 2009/06/23. doi: EC.00074-09 [pii] 10.1128/EC.00074-09 . 19542309 PMC2725557

[105] Sellam A, van het Hoog M, Tebbji F, Beaurepaire C, Whiteway M, Nantel A. Modeling the transcriptional regulatory network that controls the early hypoxic response in Candida albicans. Eukaryot Cell. 2014;13(5):675–90. 10.1128/EC.00292-13 24681685 PMC4060469

[106] Saito R, Smoot ME, Ono K, Ruscheinski J, Wang PL, Lotia S, et al A travel guide to Cytoscape plugins. Nat Methods. 2012;9(11):1069–76. 10.1038/nmeth.2212 23132118 PMC3649846

[107] Merico D, Isserlin R, Stueker O, Emili A, Bader GD. Enrichment map: a network-based method for gene-set enrichment visualization and interpretation. PLoS One. 2010;5(11):e13984 10.1371/journal.pone.0013984 21085593 PMC2981572

[108] Garcia C, Tebbji F, Daigneault M, Liu NN, Kohler JR, Allen-Vercoe E, et al The Human Gut Microbial Metabolome Modulates Fungal Growth via the TOR Signaling Pathway. mSphere. 2017;2(6). 10.1128/mSphere.00555-17 29242837 PMC5729221

