# Supplementary figures and images for: The p38/HOG stress-activated protein kinase network couples growth to division in Candida albicans
Source: PLoS Genet. 2019 Mar 28;15(3):e1008052. doi: 10.1371/journal.pgen.1008052 (PMC6456229; doi:10.1371/journal.pgen.1008052)

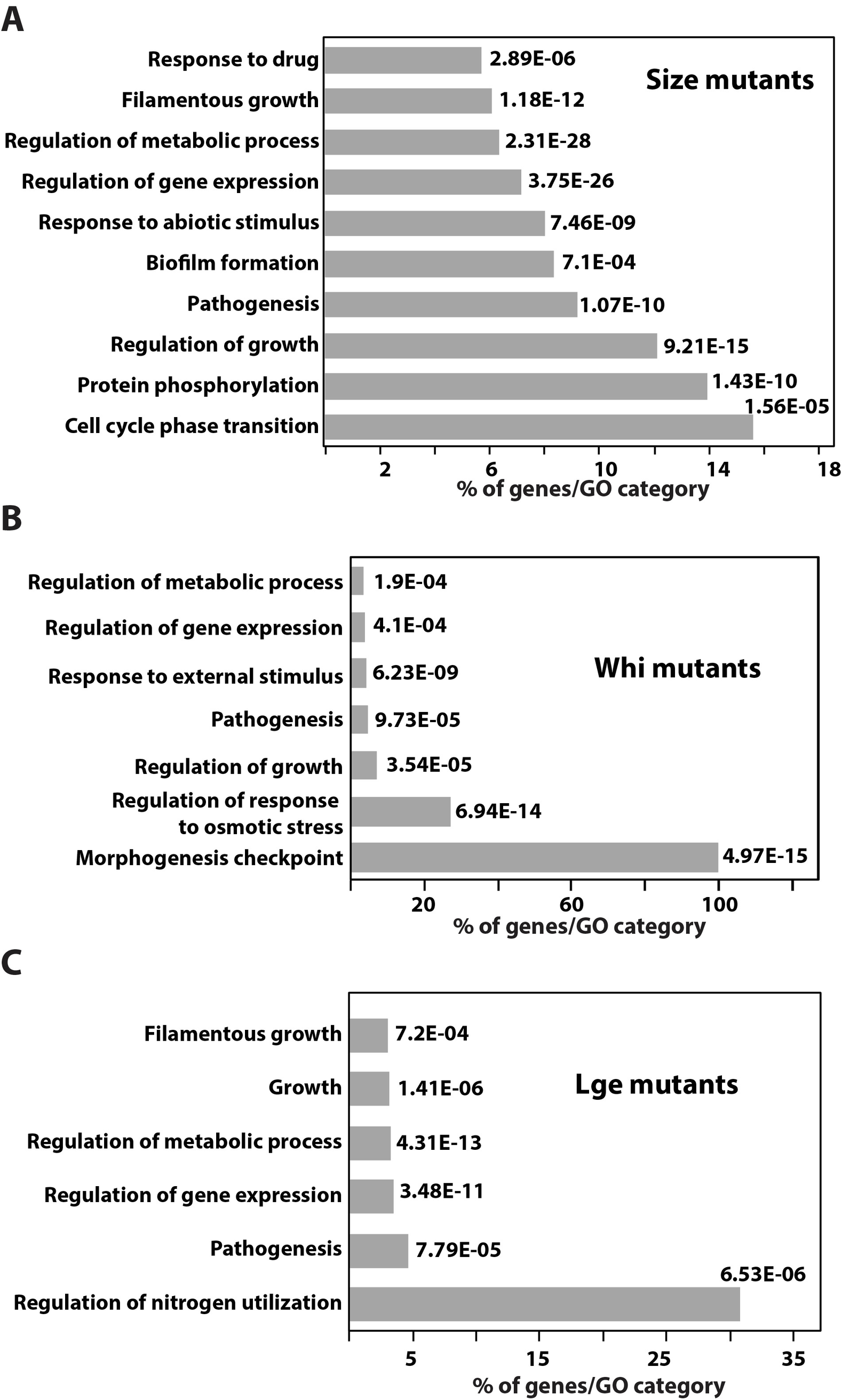

Supplement: S1 Fig — (A) GO biological process term enrichment of all 66 size mutants identified in this study. (B) GO term enrichment of small sized mutants. (C) GO term enrichment of large sized mutants. p-values were calculated based on a hypergeometric distribution (see http://go.princeton.edu/cgi-bin/GOTermFinder). (TIF) [file pgen.1008052.s001.tif]

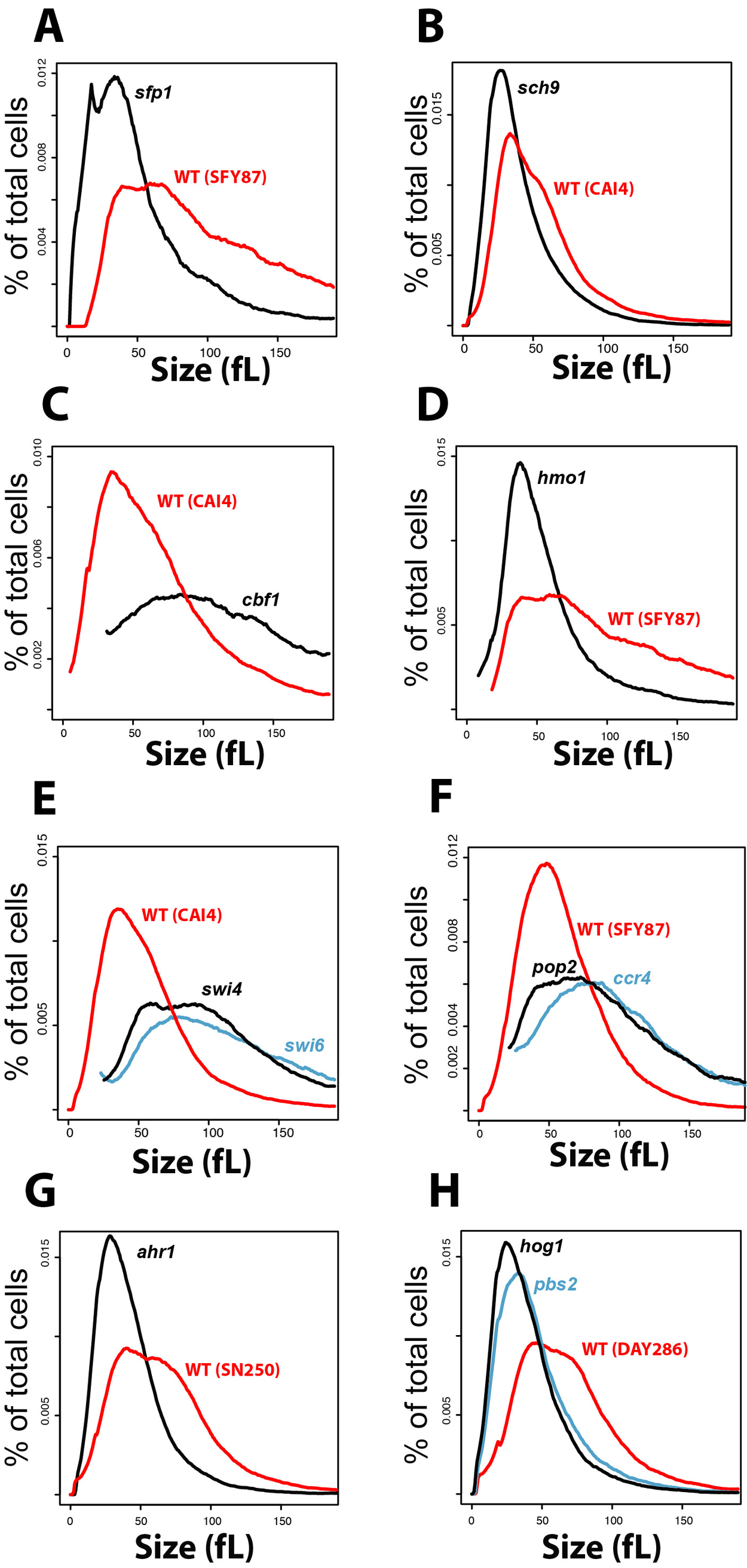

Supplement: S2 Fig — The indicated mutant strains and congenic wt control strains were grown to early log phase in rich YPD medium and sized on a Beckman Coulter Z2 Channelizer. Size distributions were shown for (A) sfp1, (B) sch9, (C) cbf1, (D) hmo1, (E) swi4 and swi6, (F) pop2 and ccr4, (G) ahr1, and, (H) hog1 and pbs2 mutants. (TIF) [file pgen.1008052.s002.tif]

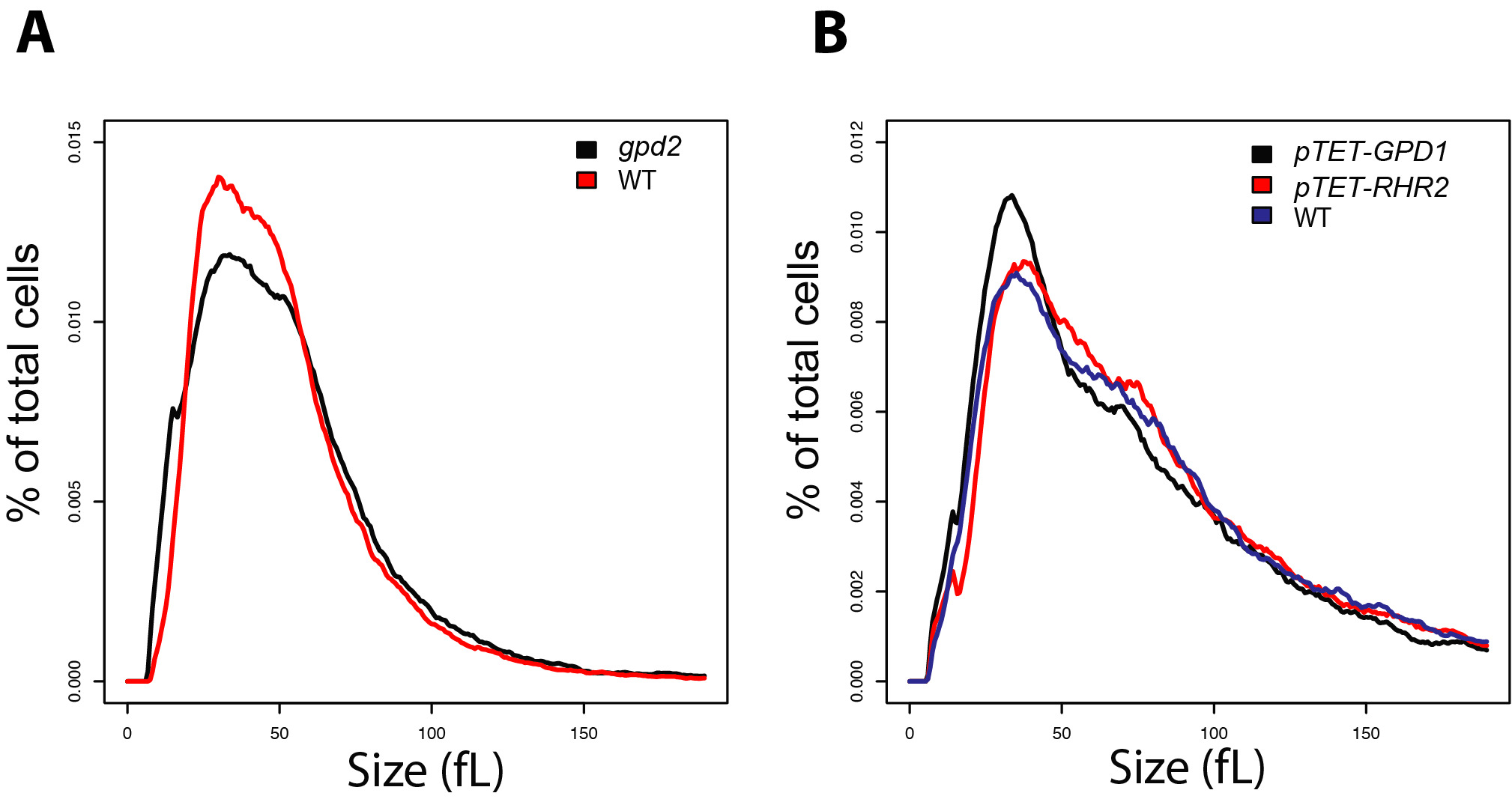

Supplement: S3 Fig — (A) gpd2 mutant and wt (SN250) strains were grown to early log phase in rich YPD medium prior to cell size determination. (B) The indicated wt (CAI4), gpd1 and rhr2 strains were grown to early log phase in rich YPD medium in the presence of doxycycline and sized on a Z2 coulter channelizer. (TIF) [file pgen.1008052.s003.tif]

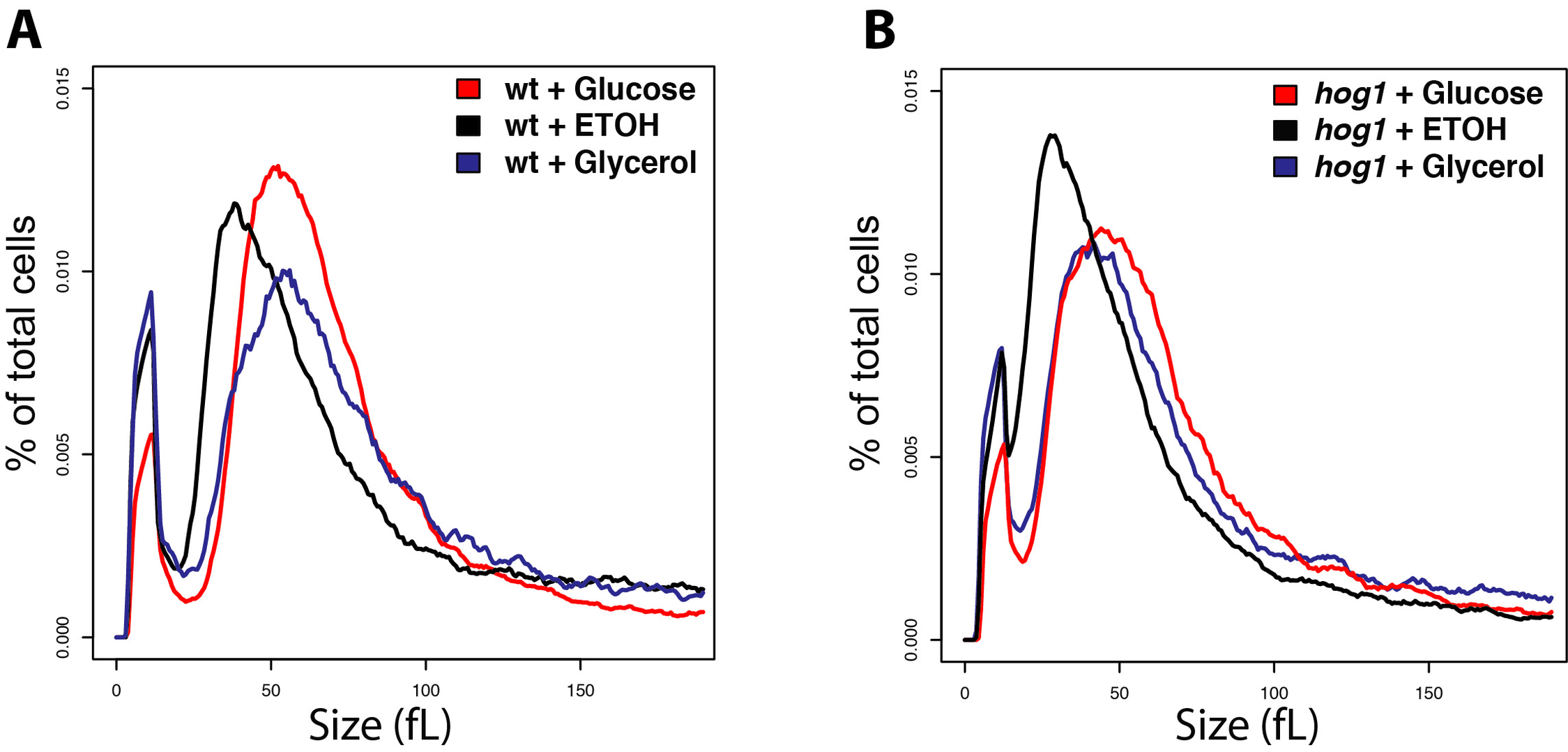

Supplement: S4 Fig — Size distribution of log-phase cultures of the indicated wt (A) and hog1 (B) strains (SN250 background) grown in synthetic glucose (red curve), glycerol (blue) and ethanol (black) medium. Cultures were sized on a Beckman Coulter Z2 Channelizer. (TIF) [file pgen.1008052.s004.tif]

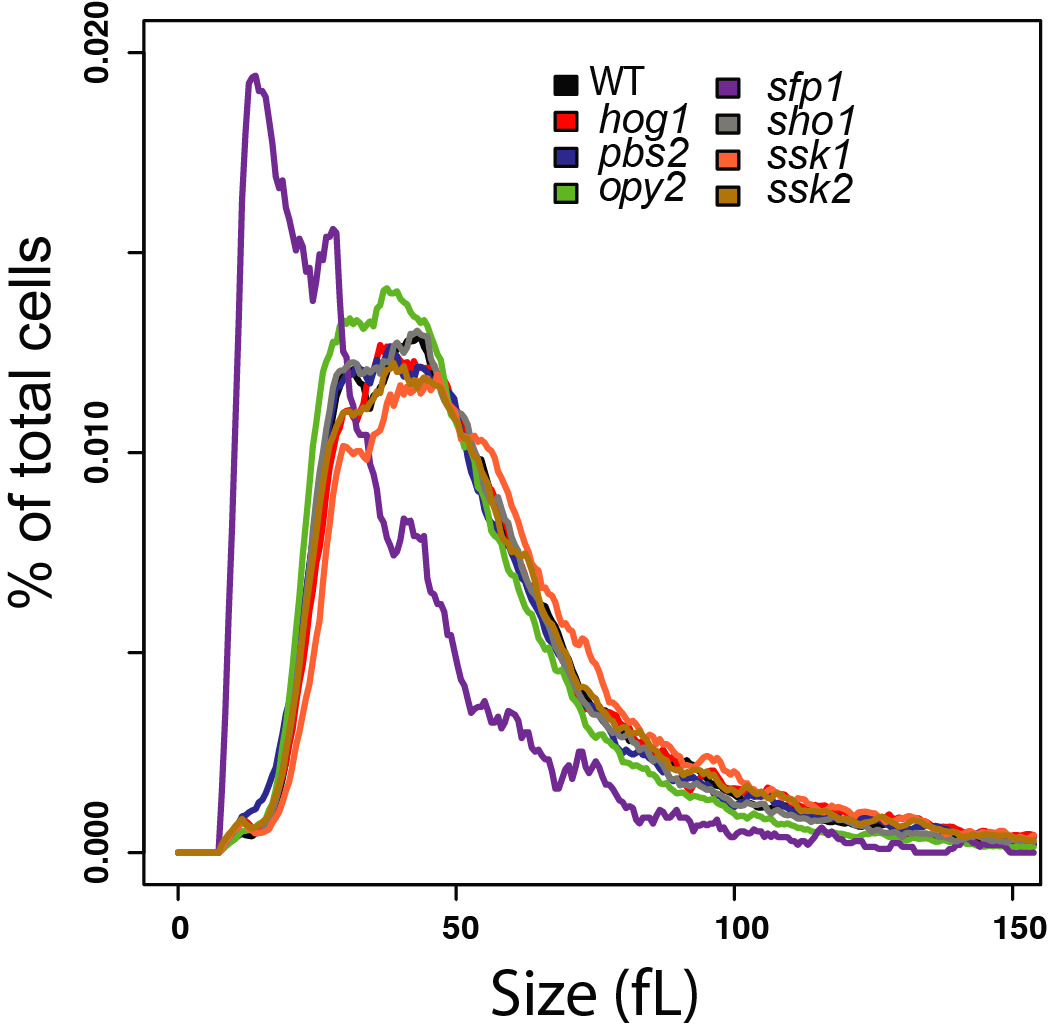

Supplement: S5 Fig — Cultures of the indicated strains were grown to early log phase in rich YPD medium and sized on a Beckman Coulter Z2 Channelizer. Wt (BY4741) and sfp1Δ strains were included as controls. (TIF) [file pgen.1008052.s005.tif]

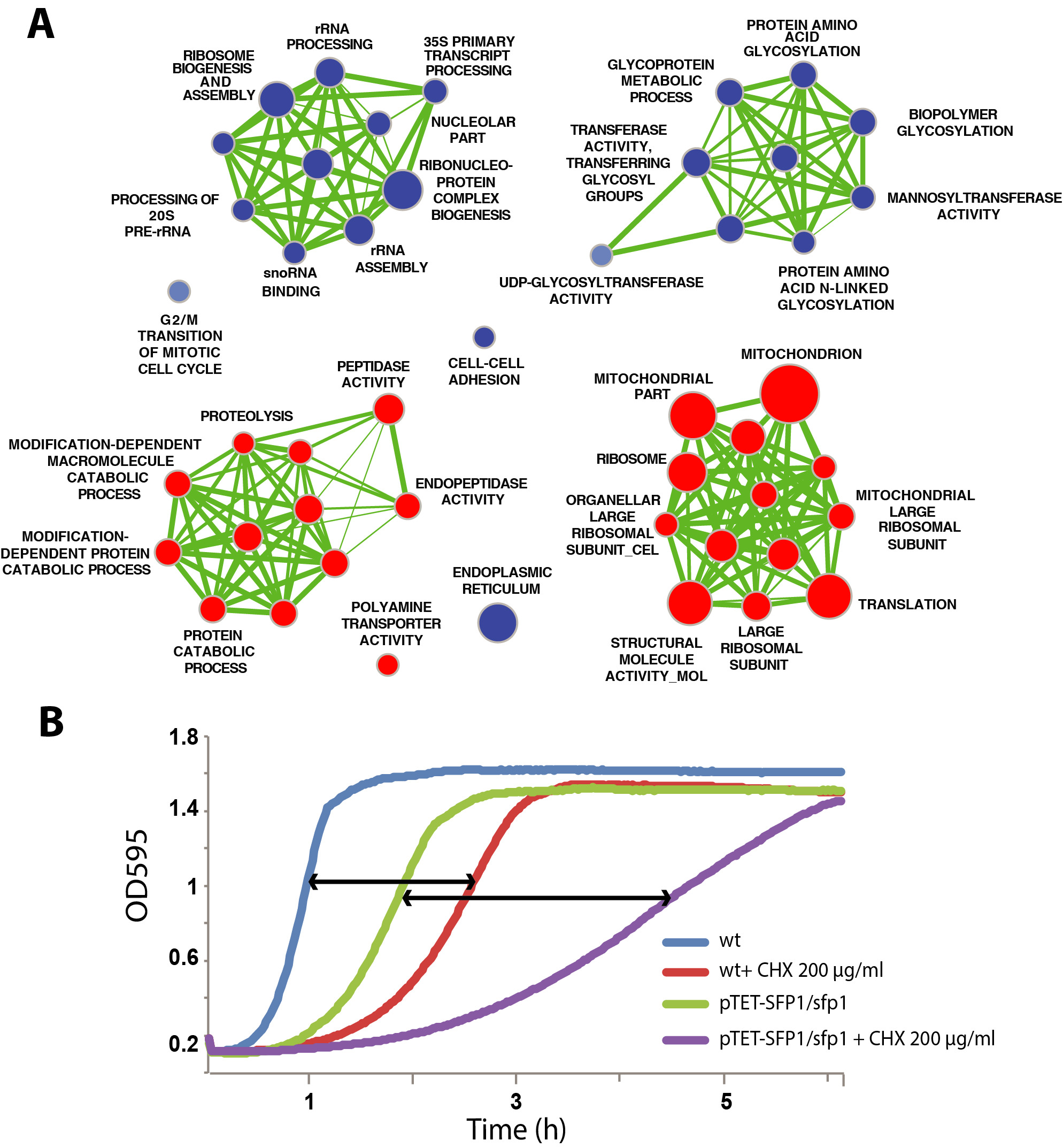

Supplement: S6 Fig — (A) Network visualization of transcriptional changes in a tet-SFP1/sfp1 conditional mutant strain. Genes expressed at reduced (blue) or elevated (red) levels after Sfp1 repression were organized into functionally connected networks (green lines) based on Gene Ontology biological process terms. Node size indicates the magnitude of change. Data were visualized using Cytoscape and the Enrichment Map plug-in. (B) A pTET-SFP1/sfp1 conditional mutant exhibited increased sensitivity to the protein translation inhibitor cycloheximide (CHX, 200 μg/ml). Cells were grown in YPD at 30°C, and OD595 readings were taken every 10 min on an automated shaker reader. (TIF) [file pgen.1008052.s006.tif]
