# Supplementary material for: The p38/HOG stress-activated protein kinase network couples growth to division in Candida albicans
Source: PLoS Genet. 2019 Mar 28;15(3):e1008052. doi: 10.1371/journal.pgen.1008052 (PMC6456229; doi:10.1371/journal.pgen.1008052)
Supplement: S1 File — (RTF) [file pgen.1008052.s014.rtf]

#####Cell size profile plotter, v1.0,#####Parameters to be modified by the user#Directory containing size files -- must be "/", not "\"##Change accordingly, to whatever folder your size files will be contained within##All coulter files with the extension below will be plottedworking.directory="working.directory"coulter.extension=".=#Z2"#File output options##Choose from 0-3 for 0=plot in R only; 1=pdf, 2=jpg, 3=tiffdesired.output=1##File name -- use valid characters only, i.e. no .,/" etc; extension will be added automatically##Files will be output in an "Output" folder created in the working directoryfile.name=“name”  ##Note:  R output is in RGB, so colors may change somewhat in typical CMYK format of most journals#Plotting parameters##Color palette to use; for names and corresponding colors, see: http://www.stat.columbia.edu/~tzheng/files/Rcolor.pdf###The length of the color palette will define how many profiles will be plotted using a defined palette###With more than 5 profiles (as presently written), colors will default to a larger randomized color palette###If more colors are added to the palette, more files can be accomodated with legends and defined palettescolor.palette=c("black","red","blue","green","purple")  ##Smoothing parameter###choose from odd numbers: 1 (no smoothing), 3, 5, 7, etc... [5-7 is usually good]points.to.average=7   ##Title and formating###Legend will only be used for small sets of profiles, with a defined palette (see above)###Long titles can be split across two lines with \n added within the title -- see example below###If you need italics, that can be done too, but it's honestly easier at that point to export as a pdf and modify in Illustratortitle="ADNtest1"line.width=2#The following parameters can be changed to increase or decrease label, axis, and title size, eg. 0.5 is half the sizelabel.scale=1axis.scale=1title.scale=1legend.scale=1#####End of parameters section#####Code for application#FUNCTIONS:#Extract size data from Coulter filespullsizedata=function(dataset)  {  diam=grep("Bindiam",dataset[,1]) #close to start of diameter measures  height=grep("Binheight",dataset[,1]) #close to start of height measures  diameters=as.numeric(dataset[(diam+1):(height-3),1])  volumes=pi*4/3*(diameters/2)^3  frequency=as.numeric(dataset[(height+1):(height+length(diameters)),1])  sizedata=data.frame(volumes, frequency)  }#Smooth datasmoothdata=function(x)  {  if(x<floor(points.to.average/2)+1)    {    output=mean(raw.frequency[1:(x+floor(points.to.average/2))])    }else if(x+floor(points.to.average/2)>length(raw.frequency)){    output=mean(raw.frequency[(x-floor(points.to.average/2)):length(raw.frequency)])    }else{    output=mean(raw.frequency[(x-floor(points.to.average/2)):(x+floor(points.to.average/2))])    }  output  }#Define color palette for large sets of size profiles##Palette will be random each time, so is only really useful for examining overall trends in size##across many profilesexclude.colors=c("grey","gray","light","white","snow")large.color.palette=colors()for(colors.i in 1:length(exclude.colors))  {  large.color.palette=large.color.palette[!grepl(exclude.colors[colors.i],large.color.palette)]  }large.color.palette=large.color.palette[sample(1:length(large.color.palette),replace=F)]#Define working directory and filessetwd(working.directory)size.files=dir()size.files=size.files[which(grepl(coulter.extension,size.files))]#Switch based on file number, determining color palette and presence/absence of a legendif(length(size.files)>length(color.palette))  {  plot.colors=large.color.palette  plot.legend=0  }else{  plot.colors=color.palette  plot.legend=1  }#Load and plot all profilesfor(file.i in 1:length(size.files))  {  temp.file=read.csv(size.files[file.i],header=FALSE,sep="\t",stringsAsFactors=FALSE)  temp.size=pullsizedata(temp.file)  temp.size=temp.size[1:(nrow(temp.size)-2),]  raw.frequency=temp.size[,2]  smoothed.frequency=apply(cbind(1:length(raw.frequency)),1,smoothdata)  frequency.fraction=smoothed.frequency/sum(smoothed.frequency)  if(file.i==1)    {    output.dir <- "Output"    if(file.exists(output.dir))      {      setwd(file.path(working.directory, output.dir))      }else{      dir.create(file.path(working.directory, output.dir))      setwd(file.path(working.directory, output.dir))      }    if(desired.output!=0)      {      if(desired.output==3)        {        tiff(paste(file.name,".tiff",sep=""))        }else if(desired.output==2){        jpeg(paste(file.name,".jpg",sep=""))        }else{        pdf(paste(file.name,".pdf",sep=""))        }      }    par(mgp=c(2,1,0),mar=c(3,3,3,1)+.1)    plot(temp.size[,1],frequency.fraction,type="l",col=plot.colors[file.i],ylim=c(0,0.015),ylab="Frequency",xlab="Size (fL)",lwd=line.width,main=title,cex.lab=label.scale,cex.axis=axis.scale,cex.main=title.scale)      setwd(working.directory)    }else{    points(temp.size[,1],frequency.fraction,type="l",col=plot.colors[file.i],lwd=line.width)    }  }  if(plot.legend==1)    {    legend(x=110,y=0.010,legend=gsub(coulter.extension,"",size.files),fill=plot.colors[1:length(size.files)],cex=legend.scale)    }  if(desired.output!=0)    {    dev.off()    }#####End of code for application
